# Rice life cycle-based global mercury biotransport and human methylmercury exposure

**DOI:** 10.1038/s41467-019-13221-2

**Published:** 2019-11-14

**Authors:** Maodian Liu, Qianru Zhang, Menghan Cheng, Yipeng He, Long Chen, Haoran Zhang, Hanlin Cao, Huizhong Shen, Wei Zhang, Shu Tao, Xuejun Wang

**Affiliations:** 1 Ministry of Education Laboratory of Earth Surface Processes, College of Urban and Environmental Sciences, Peking University, 100871 Beijing, China; 2 Department of Marine Sciences, University of Connecticut, 1080 Shennecossett Road, Groton, CT 06340 USA; 3 Key Laboratory of Geographic Information Science (Ministry of Education), East China Normal University, 200241 Shanghai, China; 4 Center for Industrial Ecology, School of Forestry and Environmental Studies, Yale University, New Haven, CT 06511 USA; 5 Finance Department, Guanghua School of Management, Peking University, 100871 Beijing, China; 6 School of Civil and Environmental Engineering, Georgia Institute of Technology, Atlanta, GA 30332 USA; 7 School of Environment and Natural Resources, Renmin University of China, 100872 Beijing, China; 8 Present Address: School of Forestry and Environmental Studies, Yale University, New Haven, CT 06511 USA

**Keywords:** Environmental sciences, Environmental social sciences

## Abstract

Protecting the environment and enhancing food security are among the world’s greatest challenges. Fish consumption is widely considered to be the single significant dietary source of methylmercury. Nevertheless, by synthesizing data from the past six decades and using a variety of models, we find that rice could be a significant global dietary source of human methylmercury exposure, especially in South and Southeast Asia. In 2013, globalization caused 9.9% of human methylmercury exposure via the international rice trade and significantly aggravated rice-derived exposure in Africa (62%), Central Asia (98%) and Europe (42%). In 2016, 180 metric tons of mercury were generated in rice plants, 14-fold greater than that exported from oceans via global fisheries. We suggest that future research should consider both the joint ingestion of rice with fish and the food trade in methylmercury exposure assessments, and anthropogenic biovectors such as crops should be considered in the global mercury cycle.

## Abstract (web summary)

Fish consumption is considered to be the only significant dietary source of MeHg. Here the authors show that rice could also be a significant global dietary source, especially in South and Southeast Asia. International rice trade and joint ingestion of fish and rice could aggravate the MeHg exposure levels in many areas.

## Introduction

Mercury (Hg) is a global pollutant and poses health risks to wildlife and humans^1^. As one of the most toxic forms of Hg, methylmercury (MeHg) can reduce the intelligence quotient (IQ) and cause developmental delays in children and may also result in cardiovascular impairment in adults^2–4^. Although Hg occurs naturally, human activities have altered its global biogeochemical cycle in the environment^5,6^. Given its long-range transport, efficient bioaccumulation in the food web, and human health impacts, global Hg cycling among various environmental media has been studied over the past several decades^7–9^. Nevertheless, most of these efforts have focused on emissions of Hg to the atmosphere, and few have examined other components, such as vegetation in the terrestrial ecosystem, in detail as well as the impacts of these components on human exposure. Recent evidence suggests that vegetation could play an important role in global Hg cycles^10^. Thus it would be desirable to identify the impacts of vegetation such as commercial crops, which could be labeled as anthropogenic biovectors, on both the global biogeochemical cycle of Hg and human exposure.

Fish consumption has been considered the single significant dietary source of MeHg in most studies^11–13^. However, this conclusion was recently challenged by studies in some rural areas in China, where elevated rice (*Oryza sativa*)-derived MeHg levels and low fish ingestion rates were reported^14^. Saturated agricultural soils, such as rice paddies, have been demonstrated to be potential MeHg production sites^15^. Although a recent paper found that human MeHg exposure across China was dominated by fish intake (including marine and freshwater products), rice consumption could be a significant dietary source of human MeHg exposure in inland China^16^. Rice is a staple food for half of the global population. However, MeHg exposure through rice ingestion has received relatively little attention compared to fish, and a global comprehensive evaluation of human MeHg exposure through rice consumption is required^14^. In addition, globalization induces a geospatial separation between the production and consumption of goods. As a consequence, unprecedented displacements of environmental and social impacts are associated with the international trade of goods^17^. Nevertheless, in many high-profile cases, the impacts of the regional and global food trades on human Hg exposure have not been suitably evaluated^18,19^. Unlike with fish, substantial rice residues, defined as the non-edible rice plant parts, are left or burned in the fields after harvest^20^. Globally, Hg emission from biomass burning contributes significantly to the Hg cycle^9^, but the contributions of different crop residues via burning have not been quantified. Thus it would be of interest to quantitatively connect global Hg cycles with human health impacts by considering the effects of anthropogenic biovectors such as rice cultivation, which is of considerable global and societal importance.

The main objective of this study is to identify and discuss the role of rice in the Hg exposure continuum (from the environment to people), including its production, residue disposition, regional trade, human MeHg intake, and potential health impacts. We first establish 27 total Hg (THg, including all forms of Hg) and MeHg inventories covering 56 years for different countries and regions, including global rice production, import, export, stock variation, domestic supplies (including food, feed, seed, processing, losses, and other uses) and human intake and potential health impacts, as well as Hg amounts in rice residue yields and those emitted from residue burning. We then evaluate the impacts of the international rice trade on domestic human MeHg exposure. We also identify human MeHg exposure through consumption of rice from different types of Hg-contaminated sites.

Here we find that rice could be a significant global dietary source of human MeHg exposure, especially in South and Southeast Asia, and globalization significantly aggravates the MeHg exposure levels in Africa, Central Asia, and Europe via the international rice trade. In addition, MeHg exposure via the joint ingestion of fish and rice is an emerging health issue in Hg-contaminated areas in Southeast Asia. This novel assessment is motivated by our recognition of the potential importance of anthropogenic biotransport in the global Hg cycle and its impact on human health.

## Results

### Mercury accumulation in rice plants and human exposure

**Fig. 1:**
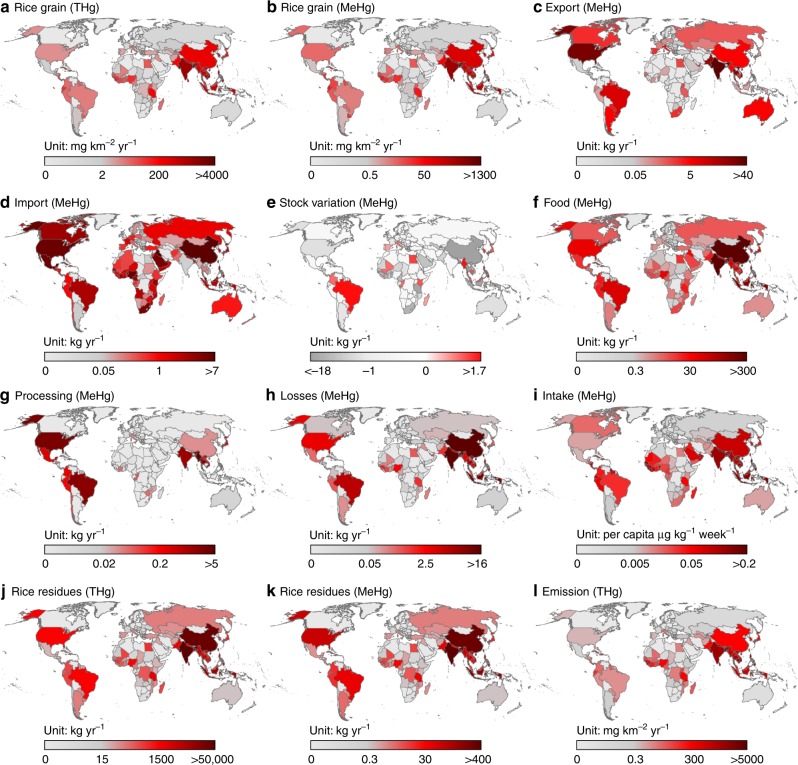
Global distribution of mercury generated during the rice life cycle. **a**, **b** Amounts of THg and MeHg generated in rice grain in 2016; **c**, **d** amounts of MeHg transported by rice export and import in 2016, respectively; **e** stock variation of MeHg in 2016; **f** amounts of MeHg supplied as food in 2013; **g** amounts of MeHg related to processing in 2013; **h** amounts of MeHg losses during transportation in 2013; **i** per capita probable weekly intake (PWI) of MeHg in 2013; **j**, **k** THg and MeHg sequestered in rice residues in 2016; **l** THg emitted from rice residue burning in fields in 2016

**Fig. 2:**
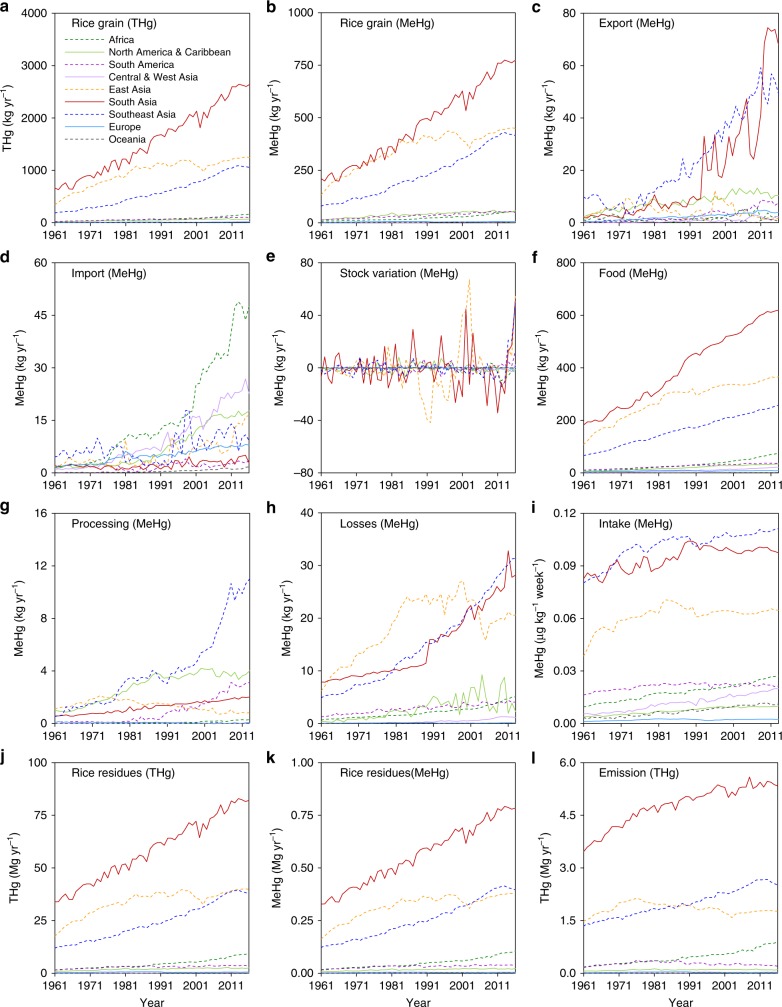
Temporal trends of mercury generated during the rice life cycle. **a**, **b** Amounts of THg and MeHg generated in rice grain from 1961 to 2016; **c**, **d** amounts of MeHg transported by rice export and import, respectively, from 1961 to 2016; **e** stock variation of MeHg from 1961 to 2016; **f** amounts of MeHg supplied as food from 1961 to 2013; **g** amounts of MeHg related to processing from 1961 to 2013; **h** amounts of MeHg losses during transportation from 1961 to 2013; **i** per capita probable weekly intake (PWI) of MeHg from 1961 to 2013; **j**, **k** THg and MeHg sequestered in rice residues from 1961 to 2016; **l** THg emitted from rice residue burning in fields from 1961 to 2016

In this study, we found that rice plants contributed a significant amount to the global anthropogenic biovectors of Hg (Figs. 1 and 2, Supplementary Figs. 1–8 and Supplementary Data 1–3). Globally, 5.3 (4.0–7.0 interquartile range from the Monte Carlo simulation) Mg of THg and 1.8 (1.5–2.2) Mg of MeHg in rice grain were harvested from terrestrial ecosystems in 2016, a substantial increase from 1.3 and 0.46 Mg, respectively, in 1961. In addition, 180 (89–360) Mg of THg and 1.7 (0.71–4.2) Mg of MeHg in rice residues were generated; both these values significantly increased from 69 and 0.66 Mg, respectively, in 1961. In contrast, 13 Mg of THg (including the edible and inedible fractions in seafood) were exported from the ocean via marine fisheries in 2014^21^. The amount of THg that was generated in rice plants (including rice grains and residues) was higher than the amount that was exported from the ocean via global marine fisheries by a factor of 14^21^. Among 281 countries and territories across the world, India (South Asia) produced the most THg in rice grain and residues (2.1 and 64 Mg, respectively, in 2016), due to its large-scale rice production and the relatively high THg concentration of rice in India^22–24^. Substantial THg was also generated in respective rice grain and residues in China (East Asia, 1.2 and 38 Mg), followed by Bangladesh (South Asia, 0.46 and 15 Mg) and Indonesia (Southeast Asia, 0.41 and 15 Mg, Fig. 1a, j). The above four countries accounted for 75% of the global THg generated by rice cultivation in 2016. Bangladesh had the highest THg production density (3.8 and 120 g km^−2^ in rice grain and residues, respectively, in 2016), followed by India (0.80 and 25 g km^−2^), Vietnam (Southeast Asia, 0.71 and 26 g km^−2^), and the Philippines (Southeast Asia, 0.62 and 20 g km^−2^), primarily due to the high population densities and the use of rice as a staple food in these countries^23^.

Among the different regions, South, East, and Southeast Asia generated most of the MeHg in rice grain in 2016, i.e., 0.77, 0.45, and 0.42 Mg, respectively, accounting for 91% of the world’s total; these values were approximately 3.7-, 3.3-, and 5.4-fold higher than that in 1961 (Fig. 2b). The generation of MeHg in rice grain in South and Southeast Asia underwent a rapid increase during the period from 1961 to 2016, while that in East Asia (mostly contributed by China, Japan, and the Republic of Korea) remained stable after 1997. The generation of MeHg in rice grain in Africa, North America (including the Caribbean), and South America has also increased rapidly in the past six decades, while the MeHg generated in Europe and Oceania substantially decreased in 1988 and 2001, respectively, but increased slowly thereafter.

**Fig. 3:**
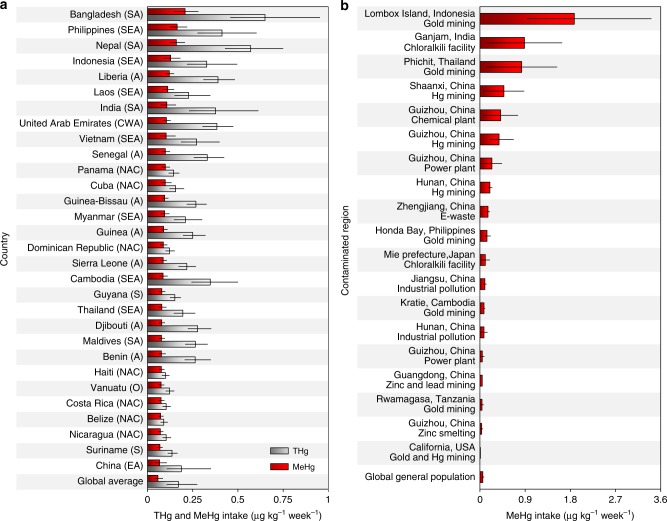
Human methylmercury intake through rice consumption. **a** Per capita probable weekly intake (PWI) of THg and MeHg through rice consumption in the top 30 countries and the world average and **b** PWI of MeHg through consumption of rice from Hg-contaminated regions. Population-weighted world average PWI values of THg and MeHg are shown. More comparison are presented in Supplementary Figs. 17 and 18. A Africa, NAC North America & Caribbean, S South America, CWA Central & West Asia, EA East Asia, SA South Asia, SEA Southeast Asia, O Oceania. Error bars in figures represent the interquartile range of the confidence intervals

We found that rice could be a significant dietary source of human MeHg exposure globally, especially in South and Southeast Asia (Fig. 1i and Supplementary Note 1). We determined that 1.4 Mg (1.3–2.0 Mg) of MeHg was domestically supplied with food in 2013, and other rice grain might contribute to significant anthropogenic biotransport of MeHg (Fig. 1c–h), which might decrease (e.g., export to partner countries and losses during transportation) or indirectly increase (e.g., import from producing countries and used as feed for livestock) the risk of human Hg exposure. Globally, the average human MeHg intake rate contributed by rice consumption was 0.057 (0.053–0.080) μg kg^−1^ week^−1^ (per capita weekly intake) in 2013 (Fig. 3a). Subsequently, 0.026 (0.012–0.047) points of per-fetus IQ decreases and 11,000 (6200–19,000) deaths from fatal heart attacks were related to the intake of MeHg through rice consumption in 2013. Interestingly, among different countries, we found that inhabitants of Bangladesh faced the highest exposure to MeHg through rice consumption (0.21 μg kg^−1^ week^−1^), followed by the Philippines (0.16 μg kg^−1^ week^−1^) and Nepal (South Asia, 0.16 μg kg^−1^ week^−1^, Fig. 3a), which are all underdeveloped countries. This situation occurred mainly due to the relatively high rice consumption rates in these countries, which were 470, 330, and 240 g day^−1^, respectively, in 2013; these values were 3.2-, 2.3-, and 1.6-folds, respectively, to the global average^23^. Subsequently, decreases of 0.10, 0.084, and 0.082 IQ points per fetus and 610, 510, and 140 deaths from fatal heart attacks in these countries, respectively, were related to the intake of MeHg via rice consumption in 2013 (Supplementary Fig. 8). Among the top 30 countries with high MeHg intake levels, 23% are in Southeast Asia and Africa.

Owing to the pressure of population growth, the portion of rice supplied as food has increased rapidly in the past six decades (Fig. 2f), especially in some underdeveloped regions. For instance, in Central Asia (also including West Asia) and Africa, MeHg exposure through rice consumption in 2013 was higher than that in 1961, by factors of 3.7 and 2.9, respectively. Southeast, South, and East Asia faced high exposure to MeHg through rice consumption, i.e., 0.11, 0.097, and 0.065 μg kg^−1^ week^−1^, respectively, in 2013. Nevertheless, we found that the MeHg intake rate was slowly increasing in Southeast Asia and peaked in 1989 and 1983 in South and East Asia, respectively (Fig. 2i). In parallel, consumption of other food (e.g., pork and poultry) has been increasing gradually^23^, which might be due to the improvement of living standards in these regions. The MeHg intake rates of inhabitants of North America and Europe through rice consumption increased in general (2.7- and 2.5-fold increases from 1961 to 2013, respectively), while the intake rates remained steady for inhabitants of South America and Oceania in recent decades.

In most regions of the world, inhabitant MeHg exposure through rice consumption would not exceed that from fish, except perhaps in special areas where rice is a staple food and is cultivated in heavily Hg-contaminated soil, e.g., gold and Hg mining areas^14,25^. Based on 1259 rice Hg measurements in 57 articles from 19 Hg-contaminated areas (Supplementary Data 4), we found that inhabitants of a gold mining area in Lombox Island (Indonesia) potentially faced the highest MeHg exposure risk through rice consumption. Assuming an inhabitant only consumes local rice^19^, the MeHg intake rate could reach 1.9 (range from 0.94 to 3.4) μg kg^−1^ week^−1^ (Fig. 3b), higher than the global general population by a factor of 33. Subsequently, 0.76 (0.34–1.5) points of per-fetus IQ decreases were related to the intake of MeHg in this area, higher than the global average by a factor of 29. MeHg intake rates through local rice consumption were also high near a chloralkili facility in Ganjam (India) and a gold mining area in Phichit (Thailand, Southeast Asia) (Fig. 3b), and 0.39 (0.19–0.73) and 0.37 (0.18–0.68) points of per-fetus IQ decreases, respectively, were related to the intake of rice MeHg in these areas. Indeed, the consumption rates of marine fish are also high in Indonesia and Thailand, which were 33 and 28 g day^−1^, respectively, in 2013, higher than the global average consumption rate by factors of 2.8 and 2.3, respectively^23^. These findings suggest that MeHg exposure through the joint ingestion of fish and rice is an emerging health issue in Hg-contaminated areas in Southeast Asia (Supplementary Note 1).

### Impacts of international rice trade and domestic economics

**Fig. 4:**
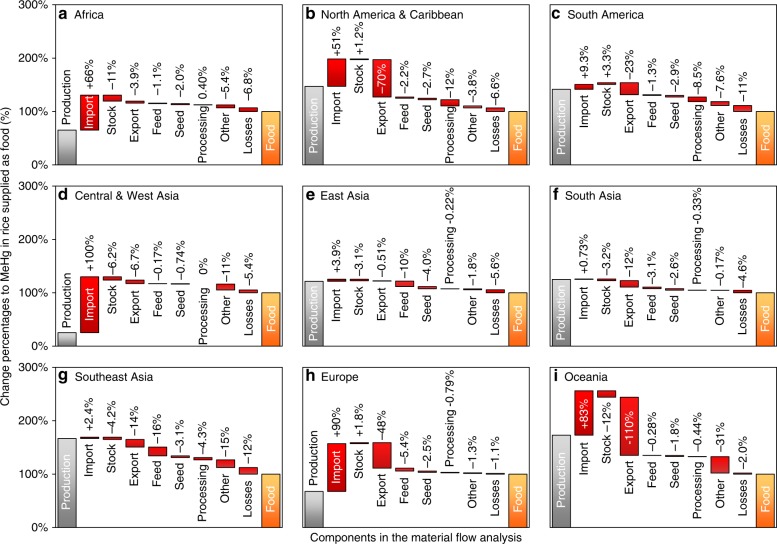
Life cycle of methylmercury generated in rice grain. Components of the life cycle of MeHg generated in rice grain include rice production, import, export, stock variation, and domestic supplies (including food, feed, seed, processing, losses, and other uses). **a**–**i** represent different regions, i.e., Africa (**a**), North America & Caribbean (**b**), South America (**c**), Central & West Asia (**d**), East Asia (**e**), South Asia (**f**), Southeast Asia (**g**), Europe (**h**), and Oceania (**i**) in the world. The life cycle of THg generated in rice grain in different regions are included in Supplementary Data 1. Data are in 2013

**Fig. 5:**
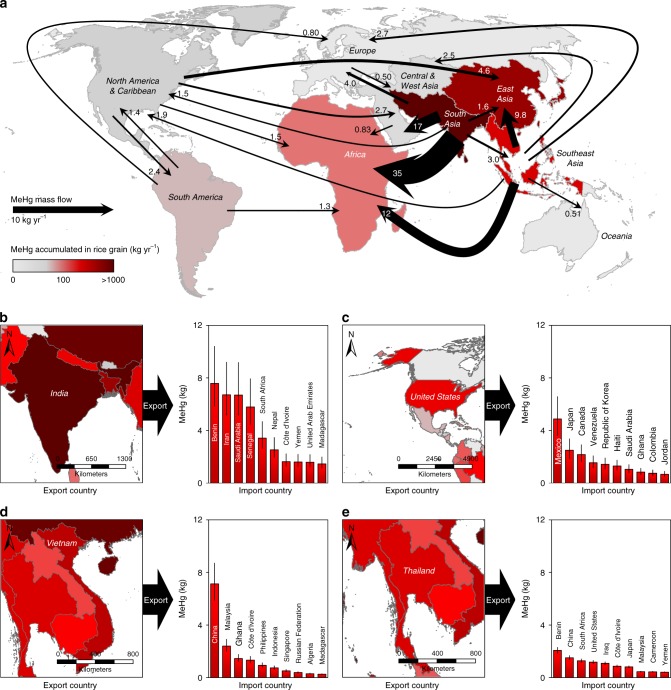
Global biotransport of methylmercury through the international rice trade. **a** shows the major MeHg flows (>0.5 kg yr^−1^) induced by the international rice trade between the regions. **b**–**e** show the top ten partner countries of the major rice MeHg-exporting countries, i.e., India (**b**), the United States (**c**), Vietnam (**d**), and Thailand (**e**), respectively. Data are in 2013. Error bars in figures represent the interquartile range of the confidence intervals

We found that the international rice trade could have significant impacts on human MeHg exposure via rice consumption in Africa, Central Asia, and Europe (Fig. 4). Globally, 9.9% of human MeHg exposure through rice consumption was embodied in the international rice trade in 2013, an increase from 3.4% in 1961. Subsequently, 2.3 × 10^−3^ (1.1 × 10^−3^–3.9 × 10^−3^) points of per-fetus IQ decreases and 710 (420–1100) deaths from fatal heart attacks were related to the international rice trade. The international rice trade aggravated MeHg exposure in Africa, Central Asia, East Asia, and Europe (increases of 62%, 98%, 3.4%, and 42%, respectively, in 2013) and mitigated exposure in North America, South America, South Asia, Southeast Asia, and Oceania (decreases of 19%, 13%, 11%, 12%, and 26%, respectively). Inhabitants of Africa consumed the highest amounts of MeHg from the trade, i.e., 35, 12, 1.5, and 1.3 kg MeHg from South Asia, Southeast Asia, North America, and South America, respectively, in 2013 (Fig. 5a). Although the amounts were lower than that in Africa, inhabitants of Central Asia also consumed 17 and 2.7 kg MeHg from South Asia and North America, respectively, in 2013.

Among the different countries, India exported the most MeHg through the international rice trade, i.e., 62 kg in 2013 (Fig. 5b), followed by the United States (23 kg, North America, Fig. 5c), Vietnam (18 kg, Fig. 5d), and Thailand (17 kg, Fig. 5e). The MeHg imports through the rice trade in India, Thailand, and Vietnam were <0.5 kg, and these countries were identified as significant net sources of global MeHg exports. Accordingly, the MeHg exposure through rice consumption for inhabitants of the four major exporters listed above was mitigated by 11%, 54%, 28%, and 24%, respectively. The total MeHg exported by these four countries accounted for 78% of the global exports in 2013. Interestingly, in contrast to other countries, the amount of MeHg generated in rice grain in the United States was not high (Fig. 1b). More than 61% of the MeHg in the United States was exported to other countries in 2013, which might be due to the low rice consumption rate in this country. In 2013, Benin (Africa), Iran (Central Asia), Saudi Arabia (Central Asia), and Senegal (Africa) consumed significant amounts of MeHg from India due to the rice trade (7.6, 6.7, 6.7, and 5.8 kg, respectively), while China also consumed 7.1 kg MeHg from Vietnam, and Mexico (North America) consumed 4.9 kg MeHg from the United States (Fig. 5b–e).

**Fig. 6:**
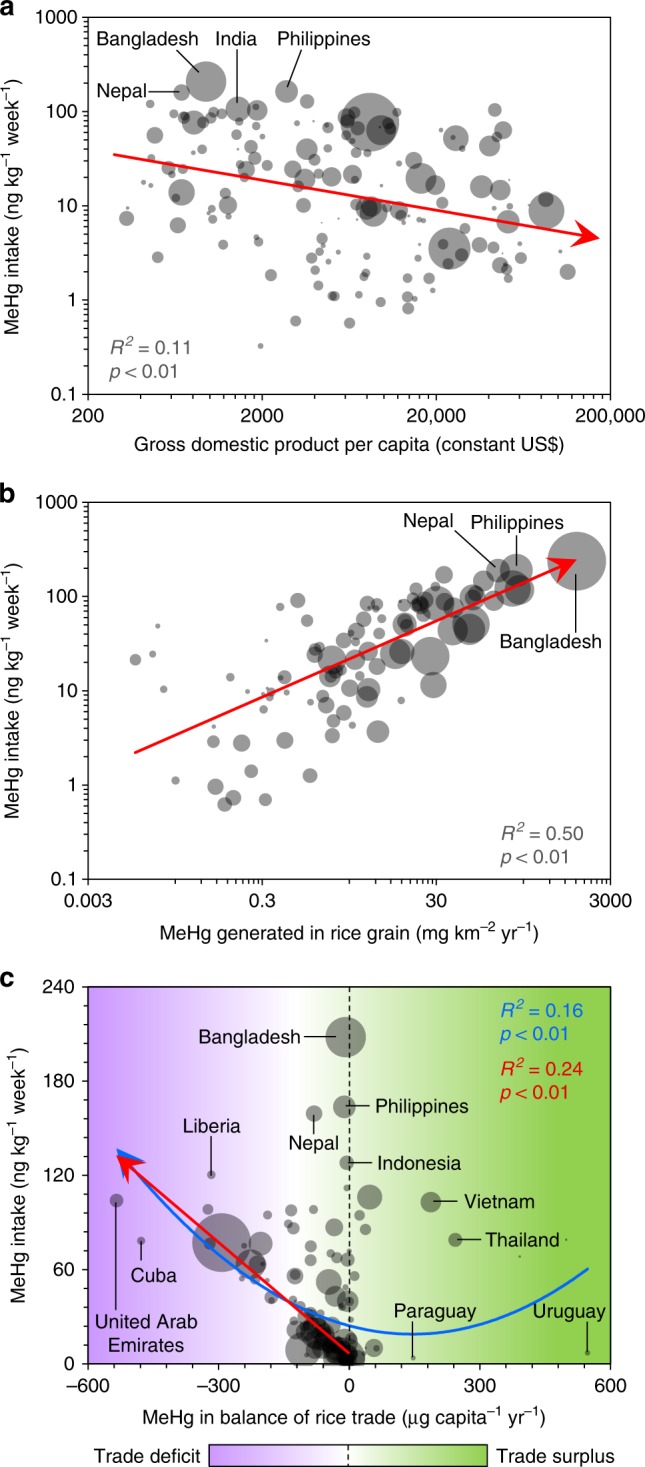
Potential driving factors of human methylmercury intake. **a** Relationship of the per capita probable weekly intake (PWI) of MeHg through rice with the gross domestic product; **b** relationship of the PWI of MeHg through rice with amount of MeHg in rice grain; **c** relationship of the PWI of MeHg through rice with the international rice trade. The gross domestic product data were obtained from the World Bank (http://www.worldbank.org/). Sample size (*n*) = 163. The sizes of the dots in the figure represent the population densities of the countries

We found that underdeveloped countries might face a relatively high level of MeHg exposure through rice consumption, while developed countries might have a lower level of exposure, based on the significant negative relationship between the MeHg intake rate and the gross domestic product of each country (*p* < 0.01, Fig. 6a). Overall, the correlation coefficient (*R* = − 0.33) was low, because at the individual level, food choices will obviously play a critical role in determining an individual’s MeHg exposure and associated risk. In addition to countries that use rice as a staple food, such as those in Asia, the relationship was also significant in other regions, such as America (*R* = − 0.36, *p* = 0.038) and Oceania (*R* = − 0.81, *p* < 0.01), but there was a lack significance in Africa (*R* = − 0.29, *p* = 0.055) and a positive relationship in Europe (*R* = 0.48, *p* < 0.01). One potential explanation is that many developed countries in Europe and North America use wheat as a staple food, and rice consumed in Europe is mainly imported from other regions (Fig. 4). Another explanation is that, owing to the improvement of living standards in many countries, consumption of meat products has gradually increased and has partly replaced the traditional staple food. For instance, the per capita consumption rate of rice in Japan decreased by 47% in the period from 1961 to 2013, while the per capita consumption rates of meat products such as pork and poultry rapidly increased by factors of 8.5 and 13, respectively, during the same period, and the consumption rate of fish products peaked in 1988^23^. The per capita consumption rates of rice in some countries, such as Brazil (South America) and China, increased initially but then decreased^23^. Future studies that could further examine this initial finding will be desirable.

There is no doubt that inhabitants of countries with a high density of rice production face a relatively high per capita MeHg intake level due to rice consumption (*R* = 0.71, *p* < 0.01, Fig. 6b). The intake is relatively low in other countries that have a high percentage of rice export and a low rice consumption rate, such as the United States. We further considered that rice import could aggravate the domestic human MeHg exposure through rice consumption (*R* = − 0.40, *p* < 0.01), especially for inhabitants of some major rice-importing countries, such as Cuba (North America), Liberia (Africa), and the United Arab Emirates (Central Asia), where the trade deficits of rice are high (Fig. 6c). For countries that have a relatively high rice consumption rate and rice production density (e.g., Bangladesh, the Philippines, Nepal, and Indonesia), the impacts of MeHg exposure caused by the international rice trade were relatively small. MeHg exposure might be different when the rice trade balance has a surplus. The exposure was low for inhabitants of Paraguay (South America) and Uruguay (South America) in 2013 because most of their rice was exported. Although Vietnam and Thailand are major rice-exporting countries in Asia, their exposure was still high because rice is a staple food in these two countries.

### Global biotransport of mercury associated with rice plants

**Fig. 7:**
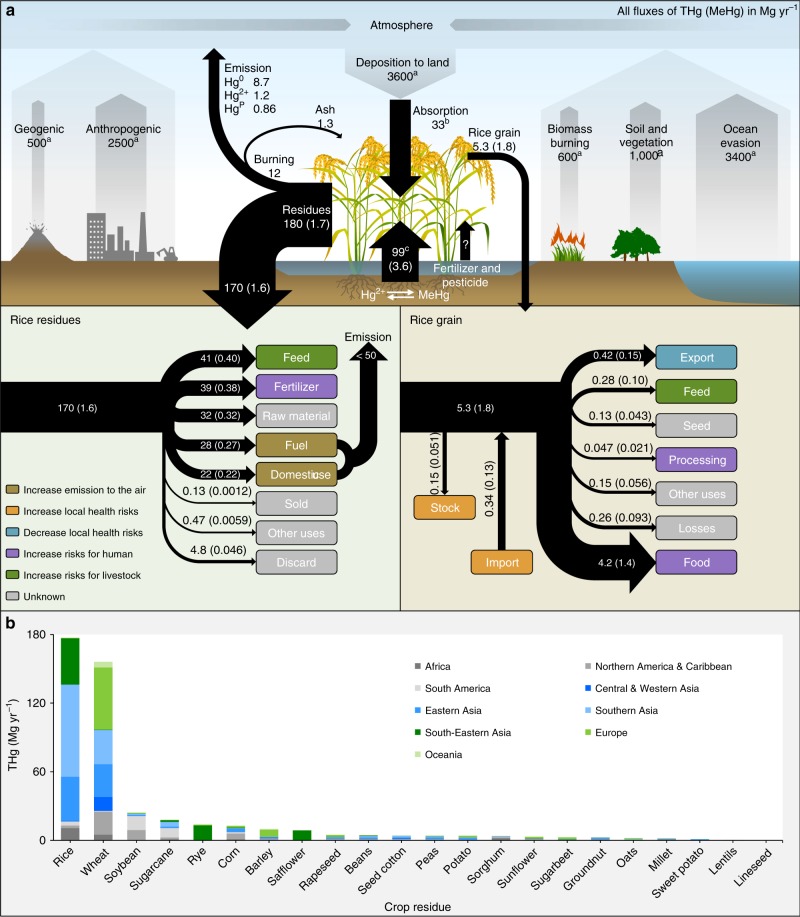
Global biotransport of mercury through rice and other crops. **a** Global biotransport of THg and MeHg through production, trade, supply, and consumption of rice grain and through different management options of rice residues; **b** global THg generated in crop residues in 2016. In **a**, fluxes of atmospheric THg emission and deposition refer to the study of Outridge et al.^31^; the flux of root absorption of THg from soil was calculated based on the rice THg uptake flux synthesized by Kwon et al.^67^; and the absorption of atmospheric Hg by rice leaves was estimated by the flux of Hg over foliage estimated by Wang et al.^68^. The global harvested area of rice was obtained from the FAO^23^

We summarized the global lifecycle of Hg associated with rice plants from its production to its final consumption (Fig. 7a). In total, 180 (94–410) Mg of THg and 3.6 (1.8–7.1) Mg of MeHg were generated in rice plants in 2013, significantly increased from 69 and 1.1 Mg, respectively, in 1961. Although the amounts of THg and MeHg generated in rice grain were lower than those in seafood, rice consumption could still be a globally significant exposure source for humans (consumed as food, 4.2 and 1.4 Mg for THg and MeHg, respectively, in 2013) and livestock (supplied as feed, 0.28 and 0.10 Mg), and possibly also for wildlife (losses during transportation, 0.26 and 0.093 Mg). According to the results of material flow analyses among different regions (Fig. 4), in 2013, 16% of MeHg in rice grain was supplied as feed for livestock in Southeast Asia, followed by East Asia (10%) and Europe (5.4%). It is advisable to further investigate whether this pathway would increase aggregate MeHg exposure. In North America, 12% of MeHg in rice grain was supplied as processed commodities and was potentially available for human intake, followed by South America (8.5%) and Southeast Asia (4.3%). Besides North America, South America, and Europe, the amount of MeHg in stocks of rice was reduced in many regions (range: −3.1% to −12%) in 2013, which were potentially used for human consumption (Fig. 1f)^23^.

Substantial Hg in rice residues is generated after harvest. Globally, 41 (20–89) and 0.40 (0.18–0.98) Mg of THg and MeHg, respectively, in rice residues were supplied as feed for livestock (Fig. 7a). Considering the substantial amounts of inorganic Hg sequestered in the stems and leaves of rice plants, Hg might present a potential risk to livestock and therefore indirectly contribute to human exposure. In addition, 39 (17–75) and 0.38 (0.16–0.95) Mg of THg and MeHg, respectively, were transported back to cropland as fertilizer, which might be a good way to return this portion of Hg to the soil. However, increasing evidence suggests that incorporating crop residues into paddy soils could enhance MeHg accumulation in rice grain^26,27^. Substantial rice residues are used for domestic purposes (some were potentially used as domestic fuel) and as industrial fuel^20,28^. Therefore, these two pathways are potential THg sources to the atmosphere (up to 50 Mg in 2016, Fig. 7a). Owing to the lack of detailed information regarding uses of rice residues as industrial and domestic fuels in different countries, their total amount and the spatial distribution of THg emissions to the atmosphere remain unknown.

In this study, we quantified that, in 2016, 11 Mg (5.2–20 Mg) of THg was emitted from rice residue burning in fields worldwide. Nevertheless, this flux might be underestimated; we discuss this issue in more detail later. Overall, India, China, Bangladesh, and Indonesia contributed 71% to the total flux (Fig. 1l). The flux of THg emissions from rice residue burning generally increased in Africa, Central Asia, South Asia, and Southeast Asia (4.8-, 6.6-, 1.6-, and 2.0-fold increases from 1961 to 2016), while it became stable in North America after 1981 and peaked in South America, East Asia, Europe, and Oceania in 1976, 1977, 1987, and 2001, respectively (Fig. 2l). The percentages of rice residue burning were typically high in Africa and Central Asia, especially in the Congo (15% in 2016), Mozambique (15%), and Gambia (14%, Supplementary Fig. 9). Outside Central Asia and Oceania, the percentages decreased over the past five decades.

We further quantified the fate of THg that was sequestered in rice residues in India, China, Thailand, and the Philippines (Supplementary Fig. 10) based on the known survey data of these countries^28,29^. These four countries contributed 62% of the global THg accumulation in rice residues in 2016 (Fig. 1j). In India, most rice residues were utilized as feed and thatching. In China, 42% of the THg in rice residues was transported back to cropland as fertilizer. A previous study showed that large amounts of rice residues were burned in the field in Thailand and the Philippines (48% and 95%, respectively^28^), and 1.4 and 2.5 Mg THg would be subsequently emitted into the atmosphere. However, data from Food and Agriculture Organization (FAO) showed that 8.5% and 7.3% of rice residues in Thailand and the Philippines, respectively, were burned in the field^23^. Similar data discrepancies also exist for India (11% and 7.5% from the literature and FAO data, respectively) and China (6.2% and 4.9%, respectively)^23,28^. Based on the literature, 17% (mass-weighted average) of THg could be released into the atmosphere through rice residue burning in these four countries, which would be 20 Mg yr^−1^, 3-fold higher than that calculated by the FAO data. In either case, we found that the present contribution of rice residue burning in the field to global THg emissions was limited, compared with 600 Mg yr^−1^ of global annual THg emissions from biomass burning^30,31^.

Motivated by the substantial THg sequestered in rice residues during cultivation, we further quantified that 460 Mg (190–1100 Mg) of THg was globally sequestered in crop residues in 2016 (Fig. 7b), 2.8-fold of that in 1961. Substantial THg (160 Mg in 2016) was also sequestered in wheat residues. This result suggests that crop residues, especially rice and wheat residues, are important biovectors induced by human activities. The fate of this portion of THg in the terrestrial ecosystem should be considered in the future.

## Discussion

Overall, our analysis showed that rice consumption could be a significant dietary source of MeHg globally, even for inhabitants of Africa, North America, and South America. Unexpectedly, countries in South and Southeast Asia, such as Bangladesh, the Philippines, and Nepal, are primary hotspots of MeHg exposure worldwide due to their high rice consumption. We found that underdeveloped countries face a relatively high level of MeHg exposure through rice consumption, and the contribution of rice consumption might be mitigated by economic growth. However, significant proportions of MeHg impacts are associated with the international rice trade, especially in Africa, Central Asia, and Europe, and this trend has risen rapidly to date. We infer that, owing to globalization, MeHg exposure induced by rice import will continue increasing for the foreseeable future.

Many modeling studies have focused on soil re-emission, assuming that plant litter would become a part of the surface soil during decomposition, and have thus neglected the role of vegetation in the global Hg cycle. Nevertheless, increasing evidence suggests that vegetation plays an important role in connecting the atmospheric and edaphic Hg cycles and emphasizes the importance of seasonal and spatial variability in vegetation uptake of gaseous elemental Hg to the global Hg balance^10,32^. We found that, at the global scale, crop plants serve as an important anthropogenic biovector and sequester a substantial amount of THg from the atmosphere and pedosphere. This component of THg could be re-emitted to the environment through burning or could be laterally transported and pose risks to other biological systems, but global Hg models have not yet been able to determine its fate. Considering that the leaves of plants sequester at least 1000 Mg of atmospheric Hg in aboveground biomass per year^33^, the Hg pools of natural vegetation and anthropogenic biovectors related to climate change and land-use change should receive detailed consideration in the global biogeochemical cycle of Hg.

The rice cultivation practices may influence THg cycling and MeHg production in paddy soil. For example, rice plants cultivated at high densities can decrease photo-demethylation of MeHg in soil^14,34^. For treatments of rice residues in different regions, burning the residues could increase atmospheric Hg emissions into the air (Fig. 7a), while the residues that degrade in paddy soils could enhance MeHg accumulation in rice grain, especially in Asia^26,27^. Globally 95% of rice acreage is cultivated under irrigated conditions^35^, while >90% of rice is cultivated in Asia^23^. Freshwater resources are stressed owing to increased water demand in Asia, and alternating wetting and drying cultivation practices have replaced continuous flooding of paddy soil since the 1970s^36^, which could lead to substantial MeHg pulses after fields are dried and re-flooded^37^. Nevertheless, an important knowledge gap remains regarding whether the alternating wetting and drying cultivation practices could lead to increased accumulation of MeHg from soil to rice grains, especially in different regions^37,38^. This is because the impacts of rice cultivation methods on MeHg accumulation in rice grain have received relatively little attention to date. In the present study, we used THg and MeHg concentrations in rice grain and residues in different regions to directly estimate THg and MeHg cycling and human MeHg intake, and different rice cultivation methods would not increase the uncertainty of the current results. Nevertheless, as suggested by a previous study, it would be desirable to further investigate the impacts of cultivation practices on MeHg accumulation in rice grain in different regions and to develop separate cultivation practices for Hg-polluted and non-polluted sites^14^.

This study represents a first attempt to quantitatively evaluate the global THg cycle and inhabitant MeHg exposure continuum through the production and trade of rice, but it has some major limitations and uncertainties. Similar to previous studies^16,18^, the age and socio-economic status of people were not considered in the MeHg intake modeling because of the difficulties in obtaining such statistical information at broad scales. However, these factors could be important to human MeHg exposure, as shown in some published studies^39,40^. Age and socio-economic status should be considered in the future, when more statistical data become available. In the present study, Monte Carlo simulation was applied to analyze the robustness of the fluxes of THg and MeHg and subsequent human health impacts, and the interquartile range was used to quantify the uncertainty. The uncertainty of results related to global and countries is provided in Supplementary Figs. 8, 11, and 12. The overall uncertainty of the results in this study is not low, especially for the amounts of THg and MeHg generated in rice residues (range: −51% to 100% and −59% to 140%, respectively) and MeHg-related health impacts (range: −50% to 67% and −39% to 56% for IQ decreases and fatal heart attacks, respectively), which are driven by the relative sparsity and large variability in measured Hg concentrations in rice. For example, as major rice-producing countries, uncertainties of amounts of THg generated in rice residues in Thailand and Pakistan ranged from −59% to 140% and from −57% to 130%, respectively. Although India potentially has high IQ decreases and fatal heart attacks associated with MeHg intake through rice consumption, substantial uncertainties existed, i.e., from −56% to 70% and from −49% to 66%, respectively. This is because Hg exposure through rice ingestion has received relatively little attention to date, particularly in geographic regions outside China^14^. This circumstance makes it difficult to accurately evaluate global Hg exposure and related health impacts through rice consumption, especially in some major rice-producing countries, such as India. It is difficult to estimate Hg accumulation in biota in a time series analysis, and thus we used modeling data to estimate the trend of Hg accumulation in rice plants, following previous studies^41,42^; this choice might have increased the uncertainty of the results. Hence, our results should be further updated when better estimation methodologies are available in the future, and future investigation of the geographic distribution of rice MeHg concentration in India and other countries is urgently needed.

At the global scale, approximately 98% of the human population could potentially consume rice, and more than half depends on rice as a staple food^23^, although MeHg exposure through rice consumption is lower than that from fish products in many regions. Indeed, the consumption rates of marine fish are also high in South and Southeast Asia, especially for inhabitants of the Philippines, Malaysia, and Indonesia^23^. We are concerned about the joint ingestion of rice as a staple food and marine fish as a major protein source in these countries because the inhabitants might be at a higher MeHg risk than the other populations of the world, which is a particular problem for MeHg-susceptible populations, such as pregnant women.

In conclusion, we present the first attempt to quantitatively evaluate the global Hg cycle and inhabitant MeHg exposure continuum via the production, trade, and consumption of rice. Our analysis indicates that a rapid increase in rice cultivation over the past six decades has resulted in substantial amounts of Hg to be accumulated in rice plants. Rice could be a significant global dietary source of human MeHg exposure, especially in South and Southeast Asia, and globalization causes significant human MeHg exposure in Africa, Central Asia, and Europe via rice consumption that is attributed to the international rice trade. In addition, MeHg exposure via the joint ingestion of fish and rice is an emerging health issue in Hg-contaminated areas in Southeast Asia. We suggest that future research should consider both the joint ingestion of rice and fish and the food trade in MeHg exposure assessments, especially in Hg-contaminated areas, and anthropogenic biovectors such as crops should be considered in the global Hg cycle.

## Methods

### Mercury generated in rice production

We first quantified THg and MeHg that were generated in rice grain and residues in each country from 1961 to 2016. The annual production of rice grain (paddy) from 1961 to 2016 was determined using the statistical data from the FAO^23^. In 2018, the FAO data provided free access to rice data for 281 countries and territories and covered all FAO regional groupings from 1961 to 2016. Some data, such as the domestic supply quantity of each country, were available until 2013. The FAO data are a pivotal tool for evaluating THg and MeHg generated in rice plants in the present study and have been previously used in a large number of studies worldwide^20,43^. To ensure that the values were comparable, we used the milled equivalents of rice grain data from the database.

Concentrations of THg and MeHg in rice grain were obtained from peer-reviewed publications, as summarized in Supplementary Data 4. In accordance with a previous study^16^, all the samples we obtained were collected in fields, from markets that were locally supplied, or imported from other countries. We excluded any data from the literature where the producing country of the rice was not provided. To make an attempt to identify human MeHg exposure through consumptions of rice from different Hg-contaminated areas, THg and MeHg concentration data from rice grown in contaminated areas were also collected in this study. A Monte Carlo simulation was applied to analyze the robustness of the fluxes of THg and MeHg and subsequent human exposure through rice consumption in the present study. To avoid the influence of any extreme values, the median values (50%) of THg and MeHg concentrations were modeled based on the Monte Carlo method. In addition, we did not consider any rice data with a sample size <3 or lacking measurement quality control in the concentration data^16^.

Globally, Hg measurements for rice grain are relatively scarce. Previous studies have used the relationships between THg and MeHg in fish and rice grain to estimate the concentrations of MeHg in the United States and China^16,18^. Based on the database we collected from published literature, we used the best fit between all THg and MeHg concentrations of rice grain suggested by the R software (version 3.3.2, R Foundation for Statistical Computing, Vienna, Austria) to model the missing THg or MeHg concentration for rice grain from non-contaminated areas (Eq. (1)) and Hg-contaminated areas (Eq. (2)) (Supplementary Fig. 13):

1 $${\rm{MeHg}} = \left( {0.80 \pm 1.1} \right) \times {\rm{THg}}^{\left( {0.65 \pm 0.072} \right)},\;R^2 = 0.46,p {\,} < {\,} 0.01^{ \ast\ast }$$

2 $${\rm{MeHg}} = \left( {0.74 \pm 1.4} \right) \times {\rm{THg}}^{\left( {0.67 \pm 0.091} \right)},R^2 = 0.61,p {\,} < {\,} 0.01^{ \ast\ast }$$

where ±SE is the standard error of the fit and is considered one of the uncertainties in the model and *R*^2^ is the correlation coefficient of the relationship. The above relationships are statistically significant (*p* < 0.01). Following the published literature^41,44^, we restricted observations to the year 2000 and later. We found that global THg and MeHg concentrations in rice grain followed a power function rather than a linear relation.

To convert the point data of THg and MeHg concentrations in rice grain into raster data and to use the data for each country or territory in the world, we applied the kriging interpolation method in this study^45^. Kriging interpolation is a useful method for estimating the geographical distribution of variables at broad scales, based on the variogram function and spatial structure analysis. Here we applied the ordinary kriging method to depict the spatial variability distribution of THg and MeHg in rice grain worldwide, and the simulation was performed using ArcGIS version 10.3. The standard errors of the interpolation results were ±8.0% and ±6.0% for THg and MeHg, respectively, and were considered in the uncertainty analysis. We also compared the measurements with our modeling results, and the comparison showed that the method was reasonable (*R*^2^ = 0.86 and 0.87 for THg and MeHg, respectively, *p* < 0.01, Supplementary Fig. 14).

Rice residues (mostly stems and leaves) are the inedible parts of the rice plant; most are left or burned in the fields after harvest. Rice residues vary widely in their properties and decomposition rates in different places. Rather than performing direct measurement, researchers prefer to estimate the mass of rice residue yield in each country based on the straw/grain ratio^20,46^. This ratio for rice is 1.5 on average, and the range of this ratio is large (0.75–2.5), which could increase the uncertainty. Rather than using this ratio, we estimated the mass of rice residues based on the FAO database of the total nitrogen content in rice residues (Mg yr^−1^) in each country. Based on the total nitrogen content in rice residues in the published literature (see Supplementary Data 5), we determined that the average total nitrogen content of rice residues was 6.5 ± 1.1‰ (average ± standard deviation).

Researchers have previously suggested that trace metal concentrations in rice grain and residues follow a linear relationship^47,48^. We examined this relationship for THg and MeHg between rice grain, stem, leaf, and residues (stem:leaf = 3: 1)^29^, based on our dataset (Supplementary Data 6) and the best relationship suggested by the R software (Supplementary Fig. 15). We found that, in most cases, relationships of THg and MeHg in different rice organs followed a power function rather than a linear relation:

3 $${\rm{Residues}}_{{\rm{THg}}} = \left( {7.5 \pm 0.34} \right) \times {\rm{Grain}}_{{\rm{THg}}} + \left( {34 \pm 38} \right),R^2 = 0.84,p {\,} < {\,} 0.01^{ \ast\ast }$$

4 $${\rm{Stem}}_{{\rm{THg}}} = \left( {4.3 \pm 1.3} \right) \times {\rm{Grain}}_{{\rm{THg}}}^{(0.92 \pm 0.063)},R^2 = 0.83,p {\,} < {\,} 0.01^{ \ast\ast }$$

5 $${\rm{Leaf}}_{{\rm{THg}}} = \left( {20 \pm 1.0} \right) \times {\rm{Grain}}_{{\rm{THg}}} + \left( {18 \pm 160} \right),R^2 = 0.91,p {\,} < {\,} 0.01^{ \ast\ast }$$

6 $${\rm{Leaf}}_{{\rm{THg}}} = \left( {2.1 \pm 1.4} \right) \times {\rm{Stem}}_{{\rm{THg}}}^{(1.2 \pm 0.067)},R^2 = 0.88,p {\,} < {\,} 0.01^{ \ast\ast }$$

7 $${\rm{Residues}}_{{\rm{MeHg}}} = \left( {0.27 \pm 1.3} \right) \times {\rm{Grain}}_{{\rm{MeHg}}}^{(1.1 \pm 0.10)},R^2 = 0.80,p {\,} < {\,} 0.01^{ \ast\ast }$$

8 $${\rm{Stem}}_{{\rm{MeHg}}} = \left( {0.31 \pm 1.3} \right) \times {\rm{Grain}}_{{\rm{MeHg}}}^{(0.85 \pm 0.12)},R^2 = 0.76,p {\,} < {\,} 0.01^{ \ast \ast }$$

9 $${\rm{Leaf}}_{{\rm{MeHg}}} = \left( {0.23 \pm 1.3} \right) \times {\rm{Grain}}_{{\rm{MeHg}}}^{(0.82 \pm 0.13)},R^2 = 0.71,p {\,} < {\,} 0.01^{ \ast\ast }$$

10 $${\rm{Leaf}}_{{\rm{MeHg}}} = \left( {0.73 \pm 1.1} \right) \times {\rm{Stem}}_{{\rm{MeHg}}}^{(0.95 \pm 0.079)},R^2 = 0.90,p {\,} < {\,} 0.01^{ \ast \ast }$$

All the fitting errors above were considered as the uncertainty of the model. If the literature provided the THg or MeHg concentrations of the stem and leaf of rice but not the bulk THg or MeHg concentration in rice residues, we calculated the Residues_THg_ and Residues_MeHg_ as follows^29^:

11 $${\rm{Residues}}_{{\rm{THg/MeHg}}} = \frac{{(3 \times {\rm{Stem}}_{{\rm{THg/MeHg}}} + {\rm{Leaf}}_{{\rm{THg/MeHg}}})}}{4}$$

We made primary estimates of the amounts of THg sequestered in other crop residues, including major cereals (excluding rice), legumes, oil crops, sugar crops, and tubers. The estimates of global crop residue production were based on the production of different crops and on research information on the straw/grain ratios of different crops (Supplementary Data 7)^20^. The THg concentrations of corn and wheat residues were obtained from Rothenberg et al. and Wang et al.^49,50^. THg measurements for other crop residues are very limited. Following the published literature^29,46^, we set the average THg concentrations (42 ng g^−1^, range: 1.0–180 ng g^−1^) for these crops based on the published data^49–53^.

It is difficult to estimate the Hg accumulation in biota using a time series analysis. Following the published literature^41,42^, we used regional Hg enrichment factors (relative to the 2010s) in soil simulated from a global box model and estimated the amount of Hg accumulation in rice plants in different regions. In the model, atmospheric Hg deposition (including anthropogenic sources and natural background) is the source of Hg in surface soil, and the trends of Hg concentrations in the soil and air in each region are the same. We compared our results with measurement data from the published literature (Supplementary Fig. 16), and the results showed that the Hg enrichment factors in soil and rice grain had similar trends.

### Biotransport of mercury through rice-related processes

Material flow analysis is extensively used as an effective tool to provide a system-oriented view of the interlinked processes of contaminants^54^. Here we used it to understand the fates of THg and MeHg in rice grain in the environment and to assess the impact of the international rice trade on domestic human MeHg exposure through rice consumption. The annual balance and trade matrix of rice grain in each country from 1961 to 2013 were determined using statistical data from the FAO^23^. In the calculation of material flow for either THg or MeHg, we considered all anthropogenic processes involving rice grain after harvest and sun-drying, including THg and MeHg in domestic export, import, stock variation, supply as feed or seed, processing, other uses, food, and losses in transportation of rice, with a final step of human exposure in each country. We established the analysis based on the mass balance principle and ensured that the amounts of THg or MeHg in the sources were equal to the amounts in the sinks, as shown below^23,54^:

12 $$\begin{array}{c}\mathop {\sum }\limits_{jk} \left[ {{\rm{Production}}_{i,j}(x) - {\rm{Export}}_{i,j}(x) + {\rm{Stock}}\;{\rm{variation}}_{i,j}(x) + {\rm{Import}}_{i,k}(x)} \right]\\ = \mathop {\sum }\limits_j \left[ {{\rm{Feed}}_{i,j}(x) + {\rm{Seed}}_{i,j}(x) + {\rm{Processing}}_{i,j}(x)} \right.\\ \left. { + {\rm{Other}}\;{\rm{uses}}_{i,j}(x) + {\rm{Food}}_{i,j}(x) + {\rm{Losses}}_{i,j}(x)} \right]\end{array}$$

where *i* represents THg (*i* = 1, kg yr^−1^) or MeHg (*i* = 2, kg yr^−1^), *j* represents each country (or reporting country in the international trade), *k* represents each partner country in the international trade, and (*x*) is the probabilistic distribution of each variable generated from the Monte Carlo simulation. Following previous studies^16,41^, we did not consider rice from Hg-contaminated sites in the material flow analysis since previous studies suggested that rice from Hg-contaminated sites was locally consumed^14,55^, and amounts of rice produced in most Hg-contaminated sites are unknown. For example, researchers found that THg and MeHg concentrations in commercial rice were generally not high in markets across China^56^. A similar situation was also found in other regions, such as Europe^24^.

To calculate THg emissions from rice residue burning in fields, the amount of THg in the burned rice residues was multiplied by the combustion efficiency using the following equation^46^:

13 $${\rm{Emission}}_{{\rm{THg}}}\left( x \right) = \mathop {\sum }\limits_j \left[ {\frac{{R_j}}{M} \times C_{{\rm{THg}},j}\left( x \right) \times E \times 10^{ - 6}} \right]$$

where Emission_THg_*(x)* is the probabilistic distribution of global THg emissions (kg yr^−1^) from rice residue burning, *R*_*j*_ is the mass of rice residue burning (Mg yr^−1^) in country *j*, *M* is the moisture content (%) of rice residues (Supplementary Data 8), *C*_THg,*j*_(*x*) is the probabilistic distribution of THg concentrations in rice residues in country *j*, and *E* is the average combustion efficiency (%) of rice residues^46^. It is challenging to quantify the fates of THg and MeHg in rice residues apart from burning due to the lack of statistical data. We made primary estimates and classified the amounts of THg and MeHg transported with rice residues as well as those left in the field and discussed associated impacts, based on existing investigation data from India, China, Thailand, and the Philippines^28,29^. These four countries contributed 62% of the global THg accumulation in rice residues in 2016.

### Domestic mercury exposure through rice consumption

Probable weekly intake (PWI) values for Hg (including THg and MeHg) were applied to evaluate the exposure through rice consumption of an individual inhabitant in each country. This method has been extensively used to estimate weekly intake of chemical contaminants^57^. Weekly human exposure to THg and MeHg was calculated based on the known THg and MeHg contents of rice grain supplied as food in each country. The calculation method is described below:

14 $${\rm{PWI}}_{ij}(x) = {\rm{Food}}_{i,j,k}(x)/P_j/{\rm{BW}}_l/52 \times 10^9$$

where PWI_*ij*_ is the probabilistic distribution of the per capita PWI of THg (*i* = 1, μg kg^−1^ week^−1^) or MeHg (*i* = 2, μg kg^−1^ week^−1^) in country *j*, Food_*i,j,k*_(*x*) is the amount of THg (kg yr^−1^) or MeHg (kg yr^−1^) in rice grain calculated from Eq. (12) that is supplied as food in country *j*, *P*_*j*_ is the population (capita) in country *j*, and BW_*l*_ is the average body weight (kg) in region *l*. In the present study, a different average body weight of the human population was used for each continent: Africa = 61 kg, Asia = 58 kg, Europe = 71 kg, North America = 81 kg, Oceania = 74 kg, and South America = 68 kg^58^. We calculated the global average PWI of THg and MeHg through rice consumption based on the population-weighted method. Because rice from Hg-contaminated sites was locally consumed^14,55^, we separately identified human MeHg exposure through consumption of rice from different types of Hg-contaminated areas, such as gold- or Hg-mining areas, areas close to smelting facilities, and other significant industrial pollution areas (Supplementary Data 4) based on Eq. (14). If the literature did not report the standard deviation of the concentration data, a 65% uncertainty was assumed^41^. Owing to lack of rice consumption rates in most of the contaminated areas in the literature, we used the rice consumption rate of the country where a contaminated area was located^19^.

### Human health impacts associated with methylmercury intake

The health impacts associated with dietary MeHg intake include neurotoxicity and cardiovascular impacts^2–4^. The neurotoxicity impact of MeHg would result in IQ decreases in fetuses, and the impact could persist into adulthood^2,59^. The association of cardiovascular outcomes and MeHg intake has been proposed for nearly two decades^60^. Nevertheless, significant uncertainties exist since inconsistent outcomes are still reported. Recently, researchers found that MeHg could diminish the cardiovascular protective effect of omega-3 polyunsaturated fatty acids and increase the risk of cardiovascular disease^61^. In addition, the United States Environmental Protection Agency has suggested sufficient evidence for the dose–response relationship between cardiovascular impacts and MeHg intake^3,18^. Following the published literature, we included fatal heart attacks as a MeHg-related health impact in the present study^18,62^. The IQ decreases and fatal heart attacks associated with MeHg intake were calculated based on the methods of Rice et al.^63^:

15 $$\Delta {\rm{IQ}}_j(x) = \gamma \times \lambda \times \beta \times \left[ {\frac{{\Delta {\rm{PWI}}_j(x) \times {\rm{BW}}_l}}{7}} \right]$$

16 $$\Delta {\rm{CF}}_j\left( x \right) = {\rm{Pf}}_j \times \omega \times \left\{ {1 - {\rm{exp}}\left[ { - \varphi \times \lambda \times \beta \times \frac{{\Delta {\rm{PWI}}_j\left( x \right) \times {\rm{BW}}_l}}{7}} \right]} \right\}$$

where ΔIQ_*j*_(*x*) represents the probabilistic distribution of the changes in IQ points in country *j*; *γ*, *λ*, and *β* are the slopes of linear relationships between the child’s IQ and the mother’s hair MeHg (IQ points per μg Hg g^−^ hair), blood MeHg (μg Hg g^−1^ hair per μg Hg L^−1^ blood), and MeHg intake (μg Hg L^−1^ blood per μg Hg day^−1^), respectively. ΔPWI_*j*_(*x*) is the probabilistic distribution of the change in the PWI of MeHg. In Eq. (16), ΔCF_*j*_(*x*) is the probabilistic distribution of the change in the number of deaths from fatal heart attacks associated with MeHg intake in country *j*; Pf_*j*_ is the number of deaths due to fatal heart attacks for people aged ≥30 years in country *j*, which was determined using statistical data from the World Health Organization (WHO). *ω* is the probability that reflects the uncertainty of the association between the hair Hg level and heart attack risk, which is a probability of one-third for the causal epidemiological associations (i.e., *ω* = 1) and two-thirds for no causal associations (i.e., *ω* *=* 0)^63^; and *φ* is the heart attack–hair Hg coefficient that reflects the relationship between hair MeHg levels and fatal heart attack risks (risk per μg Hg g^−1^ hair). The values for the coefficients *γ*, *λ*, *β*, and *φ* are based on epidemiologic studies and are referred to in the study of Rice et al.^63^, and associated uncertainties are considered in the present estimate. In the present study, we quantified IQ decreases in fetuses and fatal heart attacks in general populations associated with the intake of MeHg in rice in different countries. We also quantified the IQ decreases in fetuses associated with the intake of MeHg in rice from Hg-contaminated areas. We did not quantify the fatal heart attacks in Hg-contaminated areas since the amounts of rice produced in most of the Hg-contaminated sites are unknown. We included the time lag between MeHg intake and the response of fatal heart attacks in the estimation. The central tendency of the lag is estimated as 6 (range: 2–12) years^63^. Thus it is acceptable for the Pf data collected in the year 2016, the newest data published by WHO, to represent the impacts of MeHg intake in 2013.

### Uncertainty analysis

As described above, Monte Carlo simulation was applied to analyze the robustness of the fluxes of THg and MeHg and subsequent human health impacts, and we ran the models 10,000 times in accordance with previous studies^45^. The distribution of THg and MeHg concentrations in rice grain was confirmed to be log-normal, as found previously^16^. Because the FAO database includes official, semi-official, estimated, and calculated data^23^, uniform distributions with a fixed coefficient of deviation of 30% were set in the simulation^64,65^. Probabilistic distribution of coefficients in the health impact assessments refers to the study of Rice et al.^63^. Median values and 50% confidence intervals (interquartile range, range from 25% to 75%) of the results were generated in MATLAB (version R2017a) to quantify the uncertainties^66^. Significance was determined at the *p* < 0.05 and <0.01 levels.

## Supplementary information

Supplementary Information

Description of Additional Supplementary Files

Supplementary Data 1

Supplementary Data 2

Supplementary Data 3

Supplementary Data 4

Supplementary Data 5

Supplementary Data 6

Supplementary Data 7

Supplementary Data 8

Supplementary Data 9

Supplementary Data 10

Supplementary Data 11

Supplementary Data 12

Supplementary Data 13

Supplementary Data 14

Supplementary Data 15

Supplementary Data 16

Supplementary Data 17

Supplementary Data 18

Supplementary Data 19

## Data Availability

The authors declare that all data supporting the present study are available at the FAO website (http://www.fao.org), the World Bank website (http://www.worldbank.org/), the WHO website (https://www.who.int/), or within the article and the Supplementary Information. All data generated during this study, including source data underlying figures, are included in the Supplementary Dataset (Supplementary Data 1–19).

## Code availability

All computer codes generated during this study are available from the corresponding authors upon reasonable request.
