# Supplementary material for: Rice life cycle-based global mercury biotransport and human methylmercury exposure
Source: Nat Commun. 2019 Nov 14;10:5164. doi: 10.1038/s41467-019-13221-2 (PMC6856186; doi:10.1038/s41467-019-13221-2)
Supplement: Supplementary file 1 — Supplementary Information [file 41467_2019_13221_MOESM1_ESM.pdf]

Supplementary Information for

Rice life cycle-based global mercury biotransport and human methylmercury exposure

Liu et al.

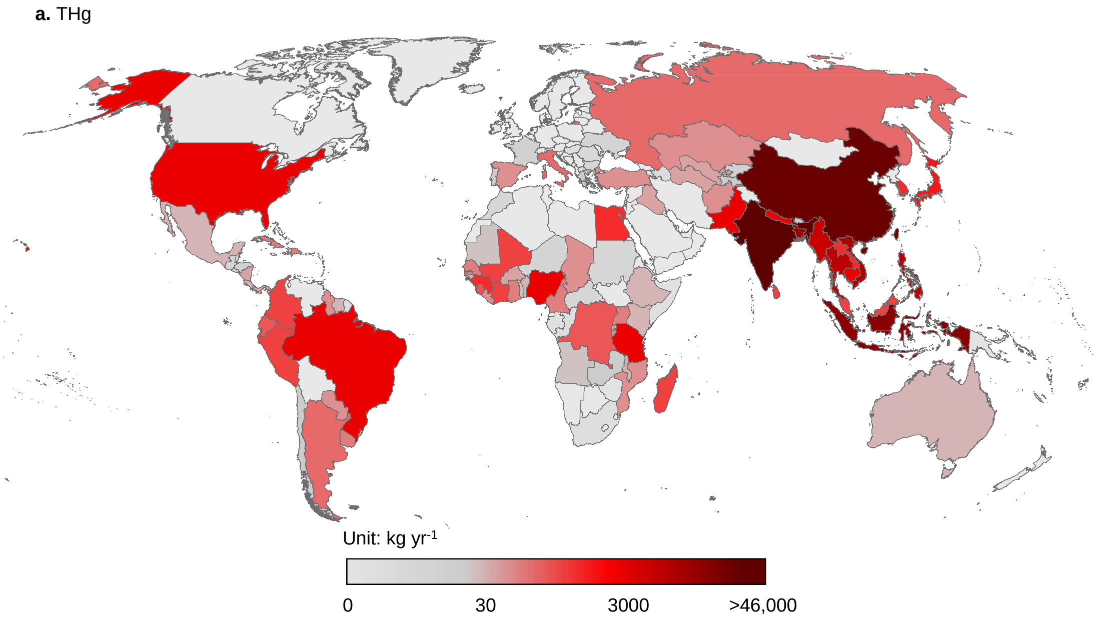

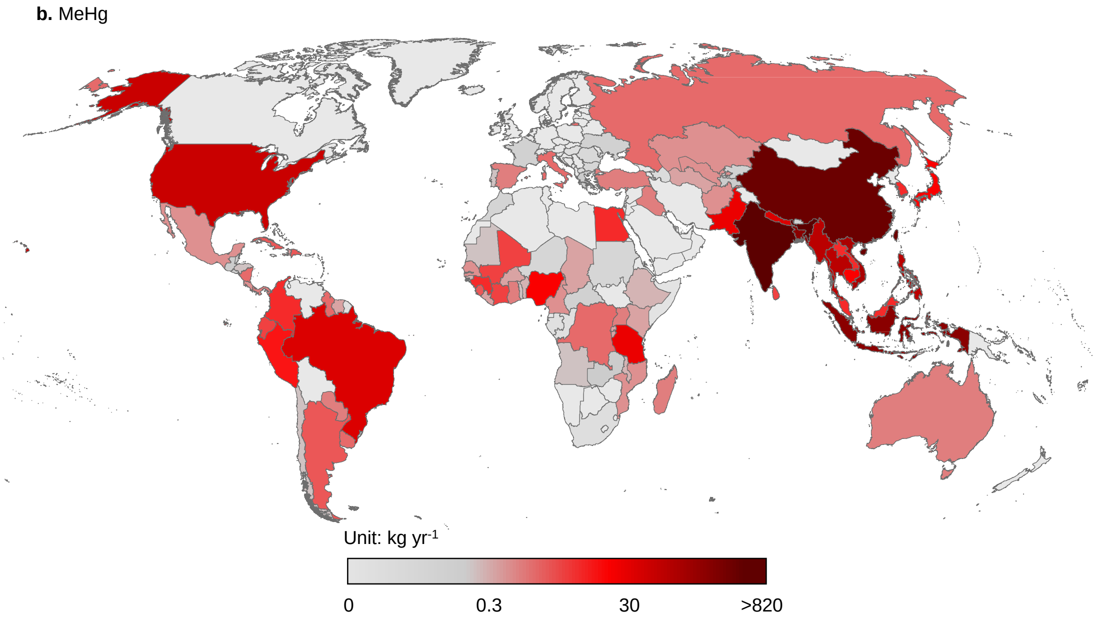

Supplementary Figure 1. Amounts of THg (panel **a**) and MeHg (panel **b**) generated in rice plant in 2016 in different countries and regions.

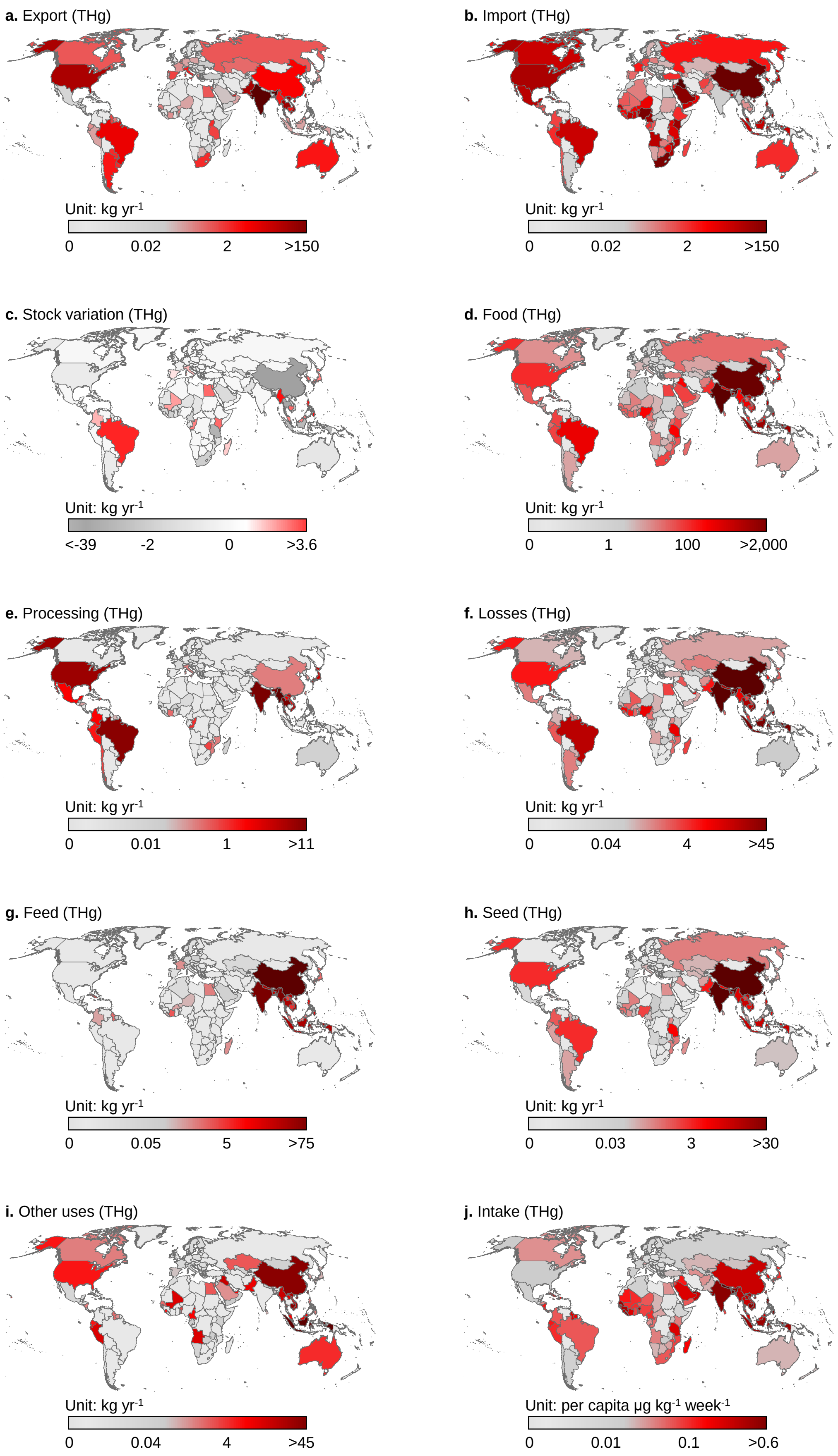

Supplementary Figure 2. Amount of THg export (panel **a**), import (panel **b**), stock

variation (panel **c**), supplied as food (panel **d**), processing (panel **e**), losses during transportation (panel **f**), supplied as feed (panel **g**), seed (panel **h**), other uses (panel **i**), and human THg intake (panel **j**) through rice in 2013.

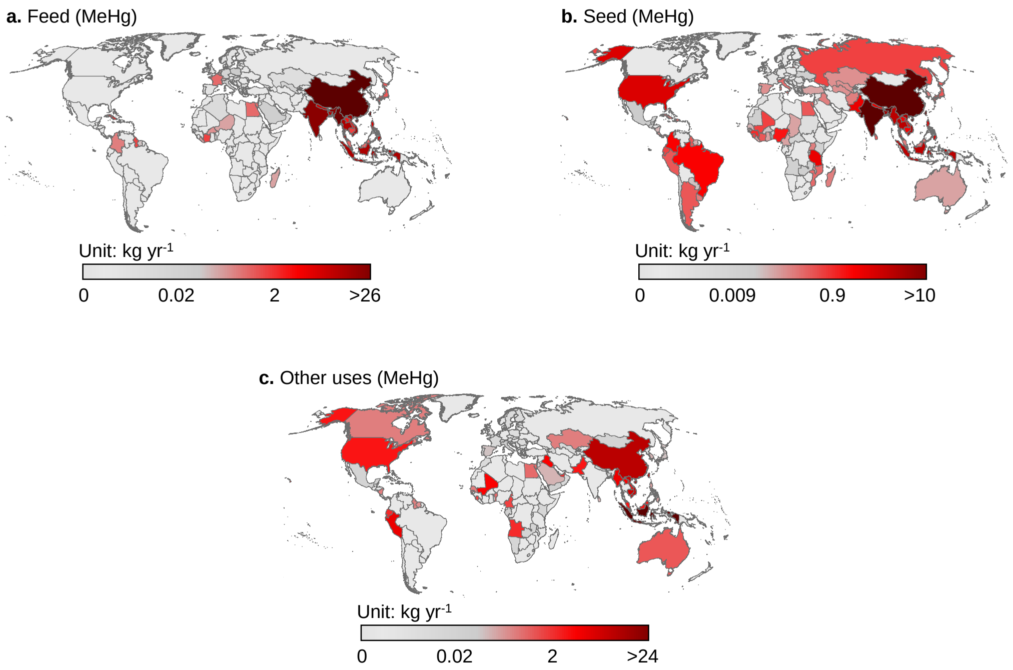

Supplementary Figure 3. Amount of MeHg supplied as feed (panel **a**), seed (panel **b**) and other used (panel **c**) through rice in 2013.

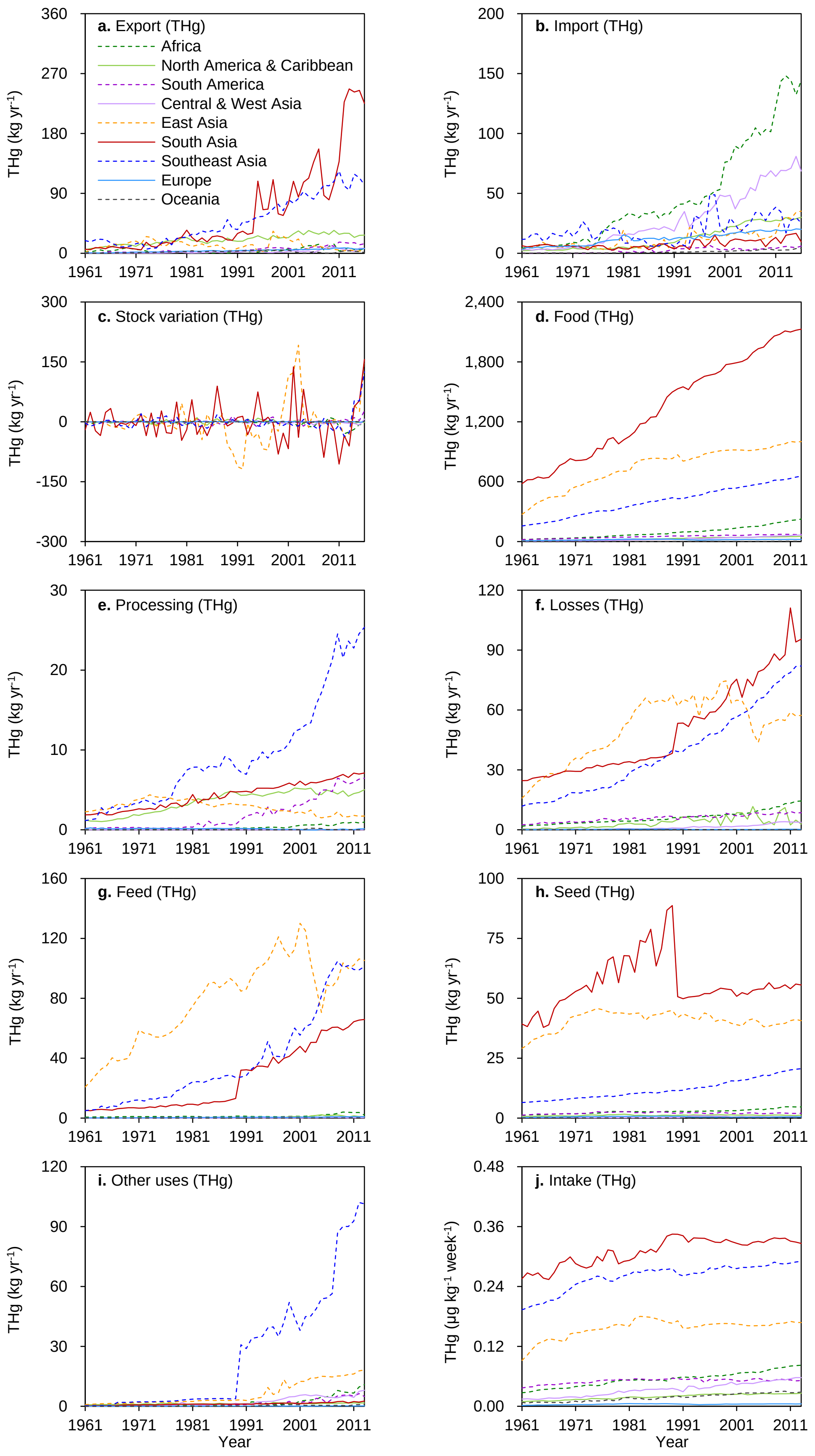

Supplementary Figure 4. Temporal trends of THg export (panel **a**), import (panel **b**),

stock variation (panel **c**), supplied as food (panel **d**), processing (panel **e**), losses during transportation (panel **f**), supplied as feed (panel **g**), seed (panel **h**), other uses (panel **i**), and human THg intake (panel **j**) through rice**.**

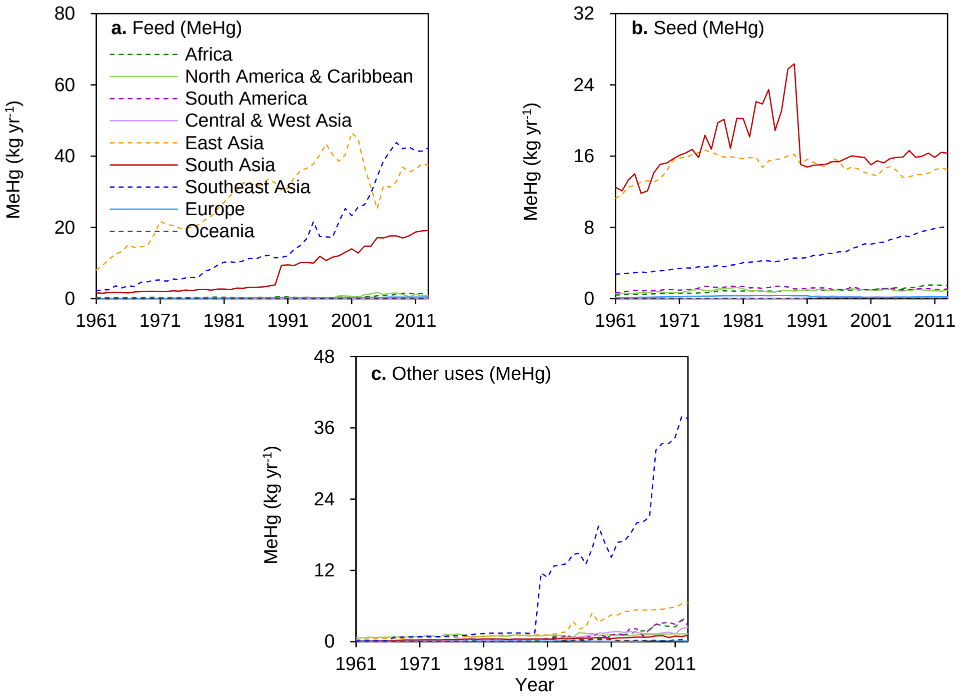

Supplementary Figure 5. Temporal trends of MeHg supplied as feed (panel **a**), seed (panel **b**) and other used (panel **c**) through rice in 2013.

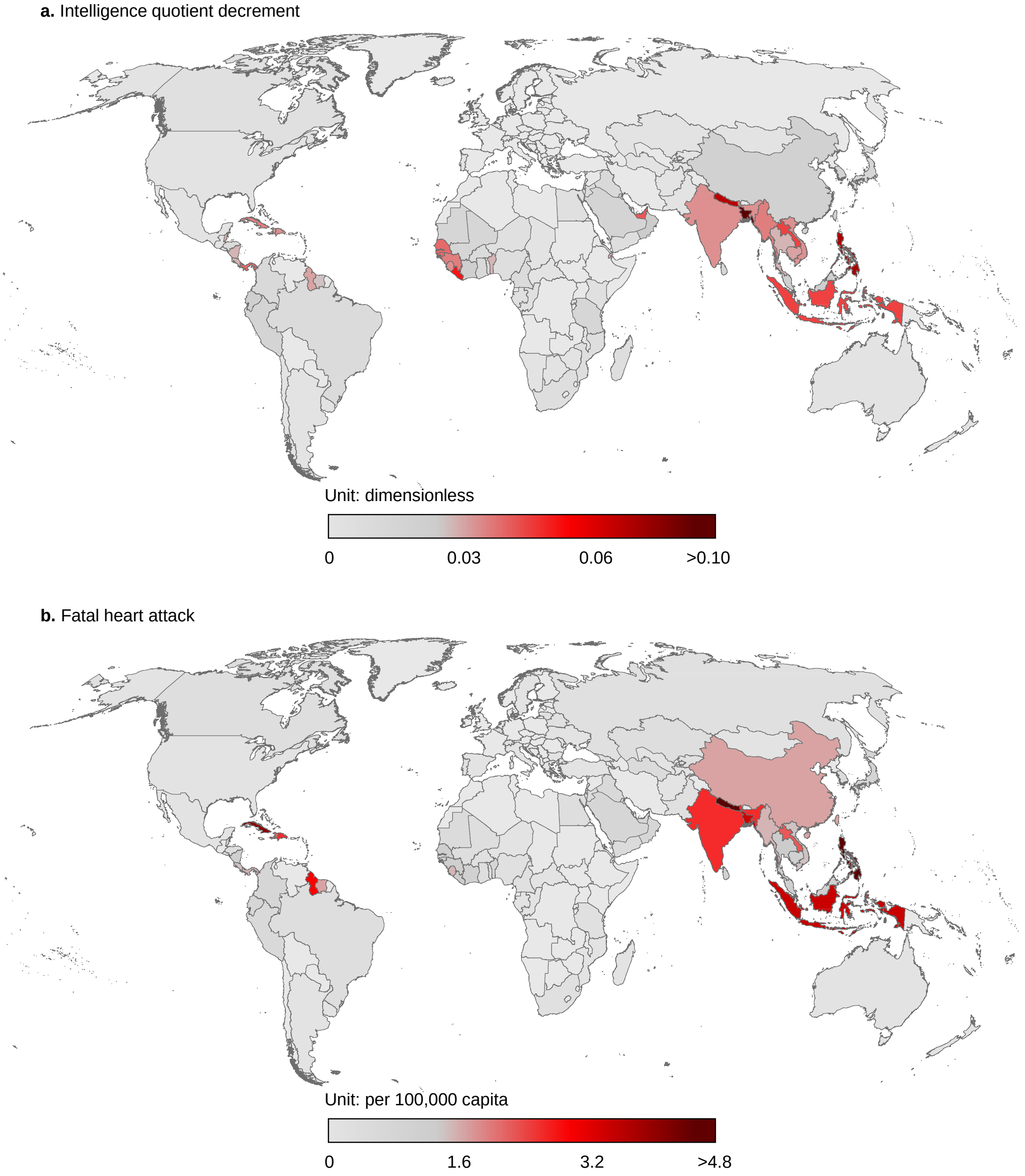

Supplementary Figure 6. Per-foetus intelligence quotient decrements (panel **a**) and deaths from fatal heart attacks (panel **b**) related to the intake of MeHg through rice consumption in 2013.

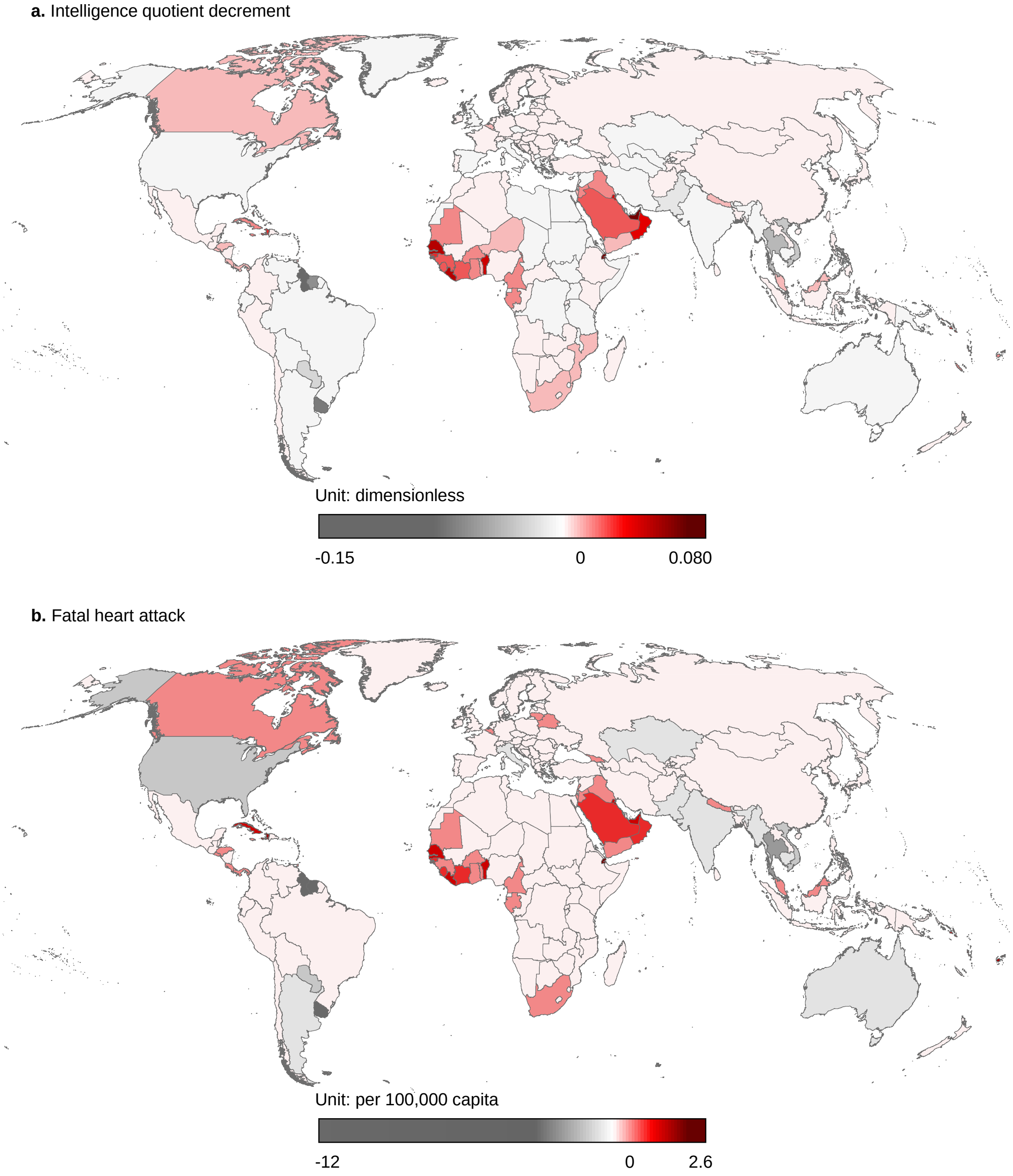

Supplementary Figure 7. Changes of per-foetus intelligence quotient decrements (panel **a**) and deaths from fatal heart attacks (panel **b**) related to the intake of MeHg through international rice trade in 2013.

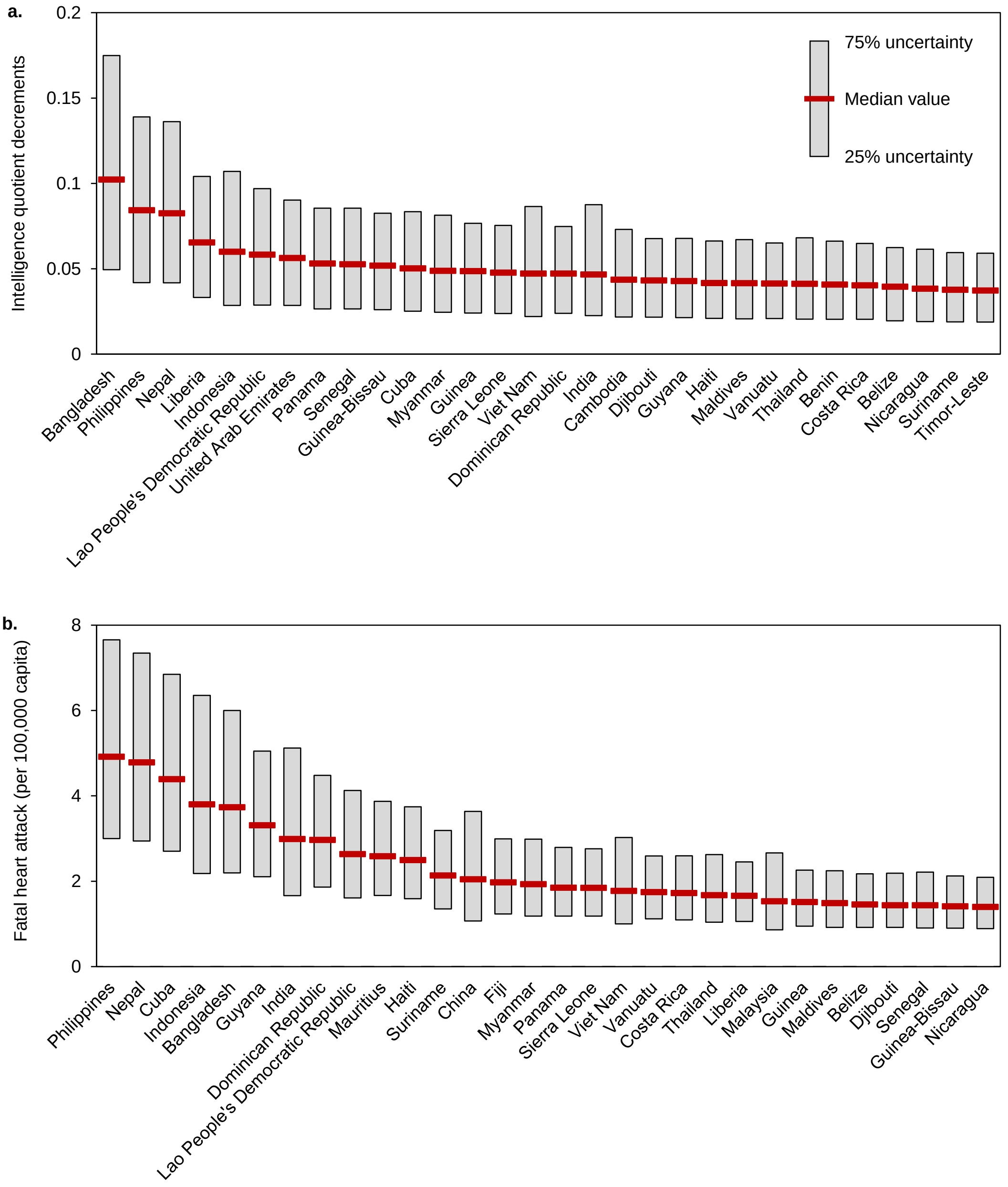

Supplementary Figure 8. Uncertainties of per-foetus intelligence quotient decrements (panel **a**) and deaths from fatal heart attacks (panel **b**) related to the intake of MeHg through rice consumption among different countries and regions.

a. Percentage of burning (spatial pattern)

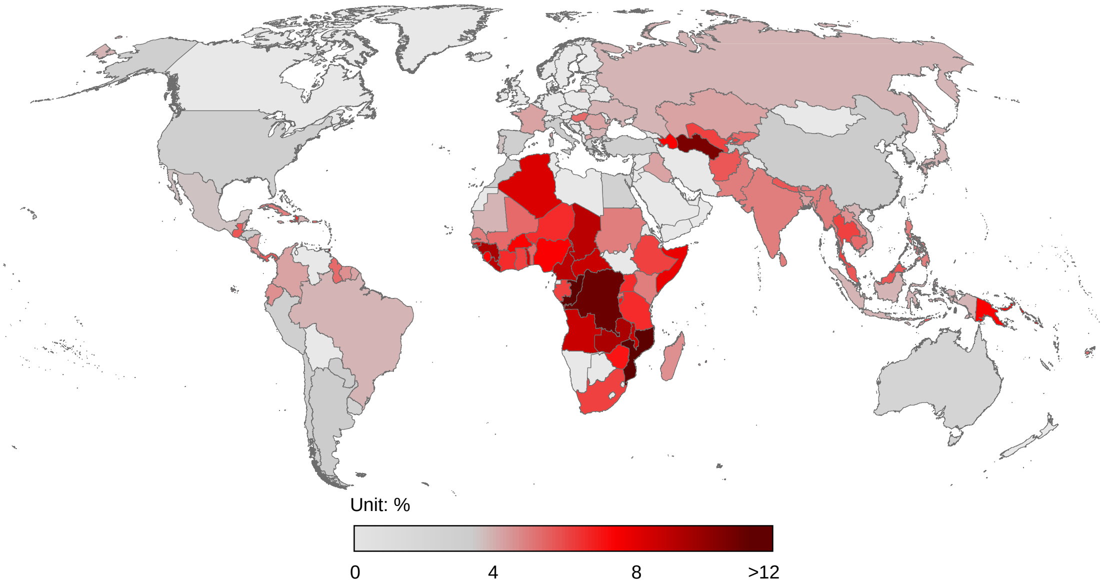

b. Percentage of burning (temporal trend)

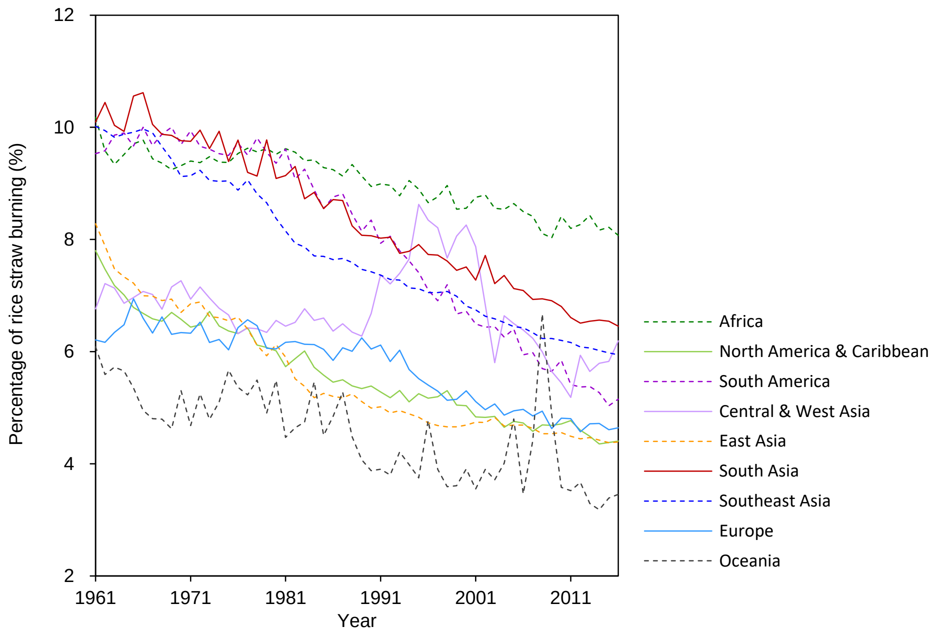

Supplementary Figure 9. Percentage of rice residue burning (panel **a**) in 2016 and temporal trends (panel **b**).

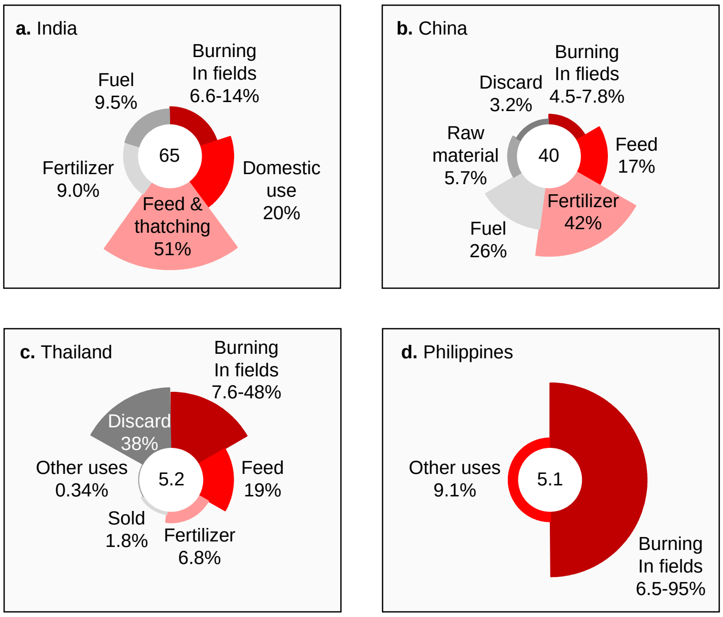

Supplementary Figure 10. Fates of THg in rice residues in India (panel **a**), China (panel **b**), Thailand (panel **c**) and Philippines (panel **d**) in recent years, respectively.

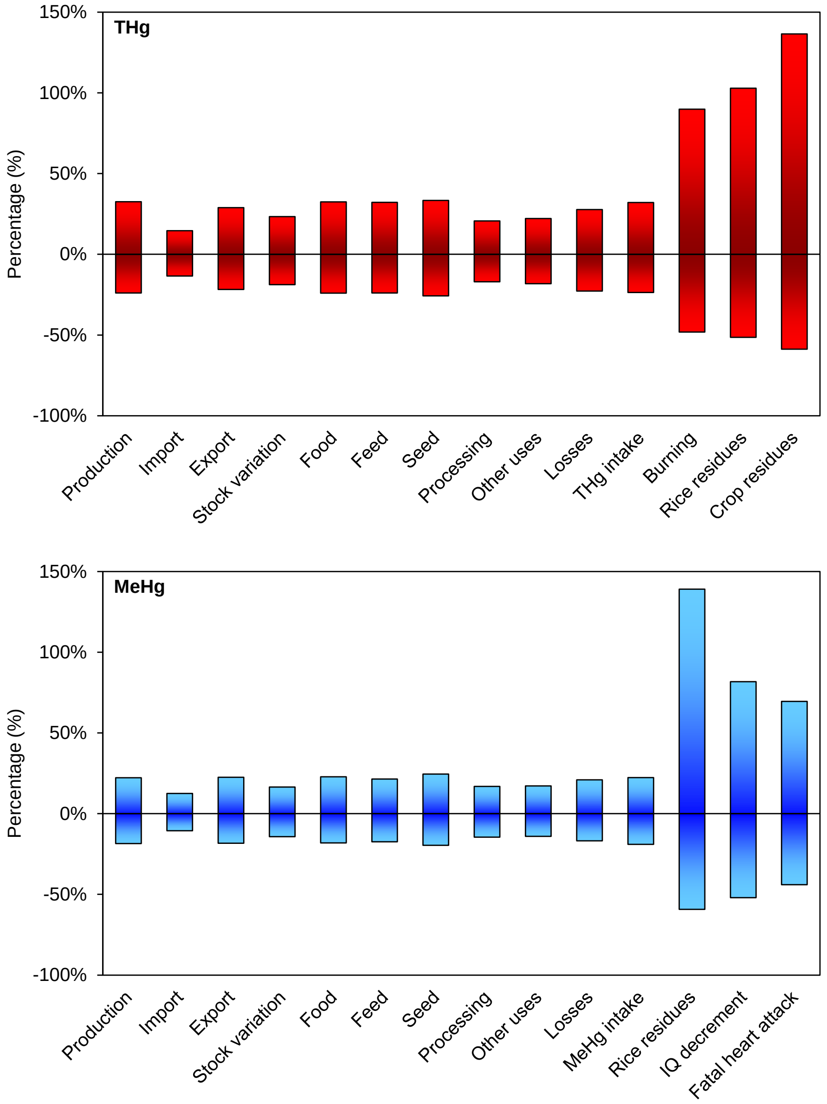

Supplementary Figure 11. Uncertainties of biotransports of THg and MeHg from production to consumption of rice grain, and generated in rice residues.

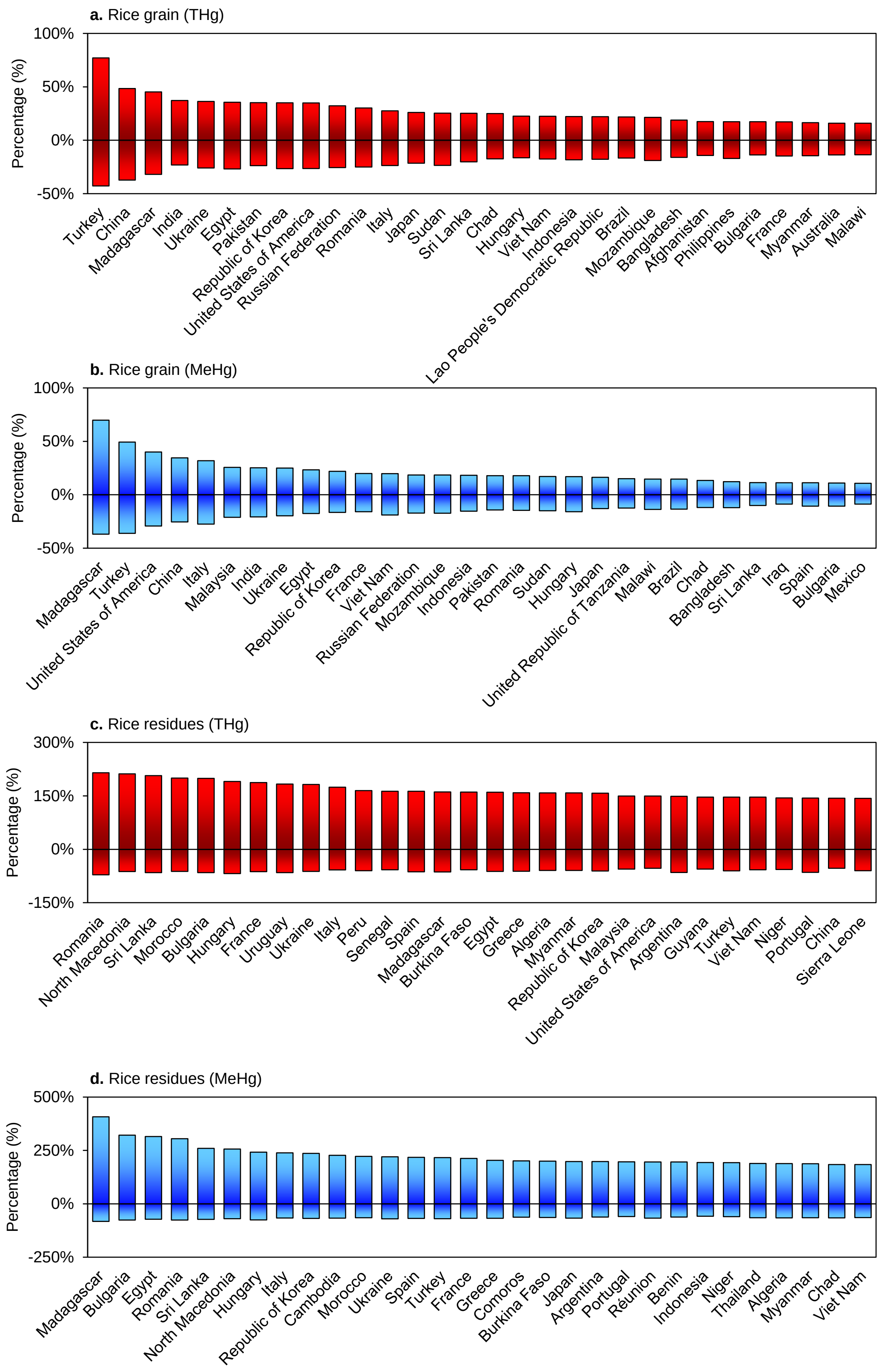

Supplementary Figure 12. Uncertainties of THg (panels **a** and **c**) and MeHg (panels **b** and **d**) in rice grain (panels **a** and **b**) and residues (panels **c** and **d**) in different countries and regions.

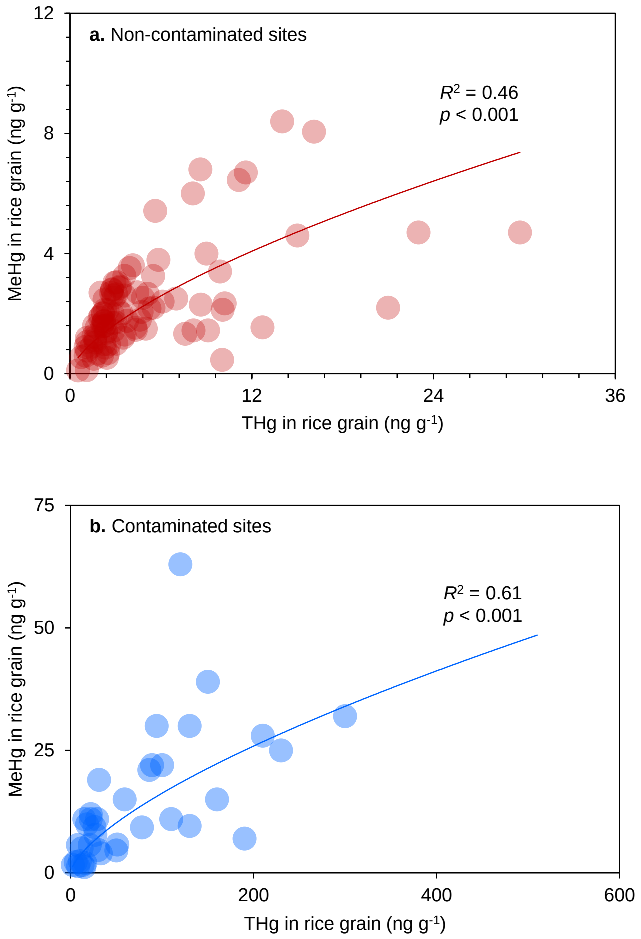

Supplementary Figure 13. Relationship of THg and MeHg in rice grain in non-contaminated sites (panel **a**) and Hg-contaminated sites (panel **b**). Sample size (n) for panels **a** and **b** are 100 and 54, respectively.

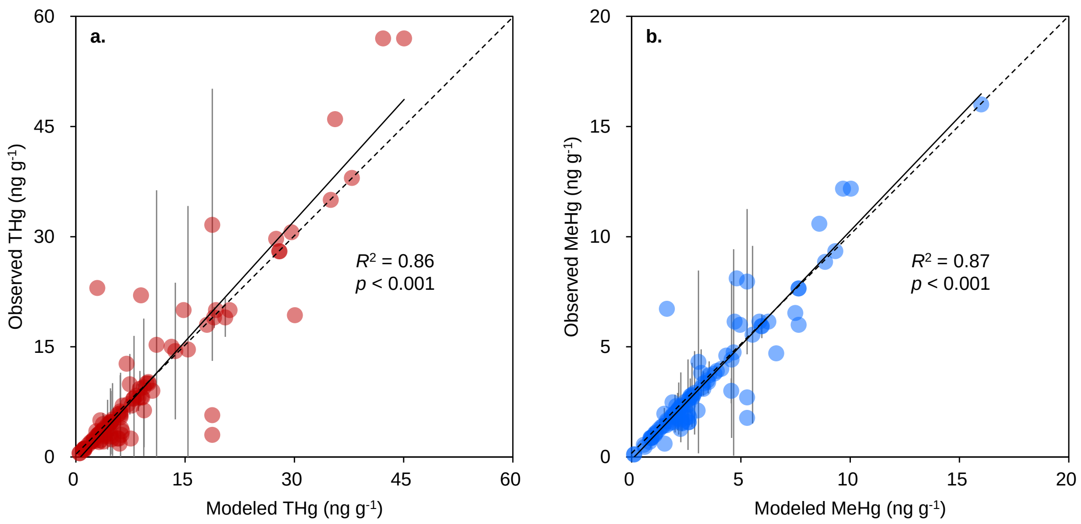

Supplementary Figure 14. Relationship of modeled results and observed data of THg (panel **a**) and MeHg (panel **b**) concentrations in rice grain, respectively. Error bars in figures represent standard deviations of different observed data in the same country/region. Sample size (n) = 100.

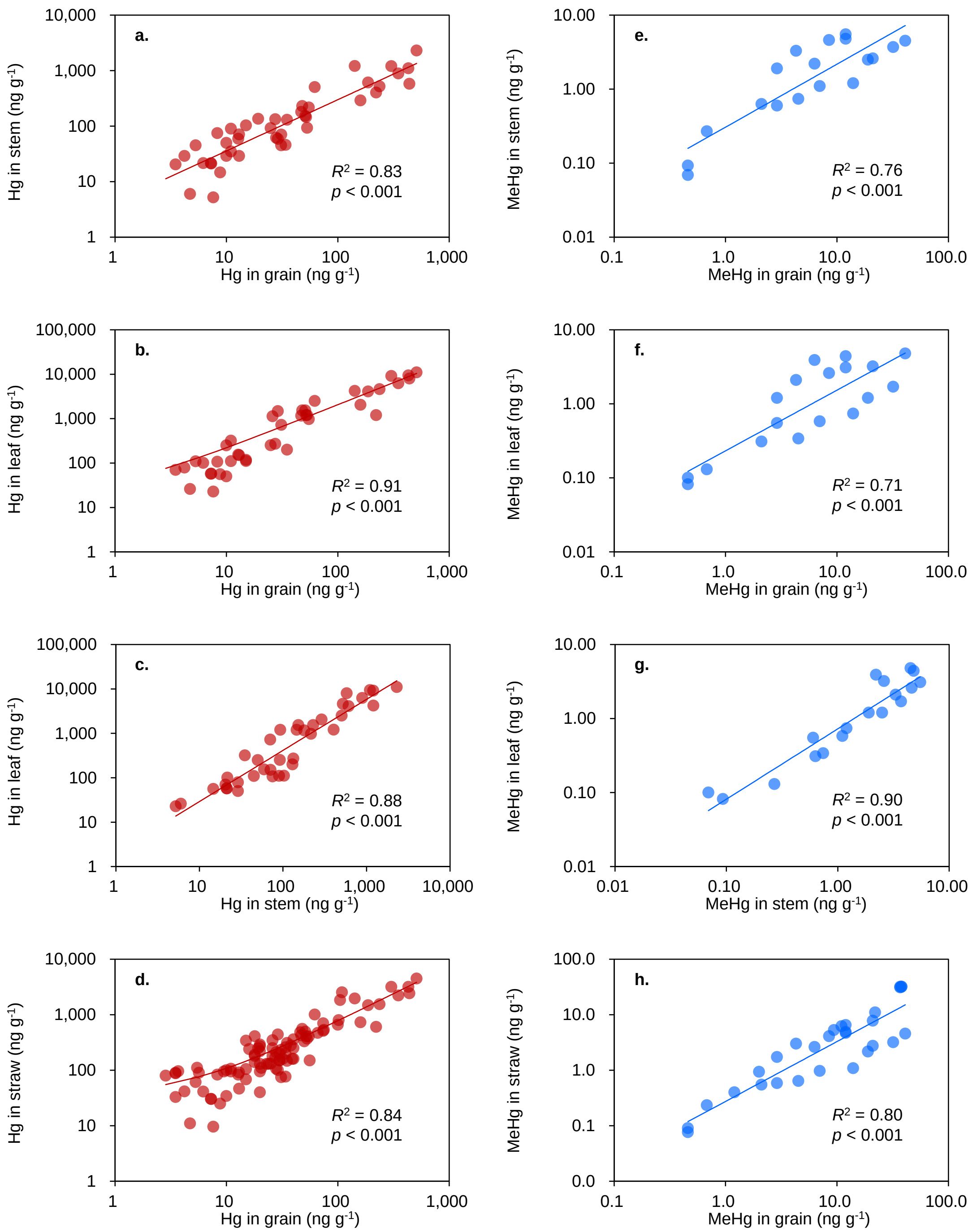

Supplementary Figure 15. Relationship of THg and MeHg concentrations in different part of rice plants. Panels **a** (sample size n = 48) and **e** (n = 19): grain and stem; panels **b** (n = 48) and **f** (n = 19): grain and leaf; panels **c** (n = 48) and **g** (n = 19): stem and leaf; and panels **d** (n = 96) and **h** (n = 30): grain and straw.

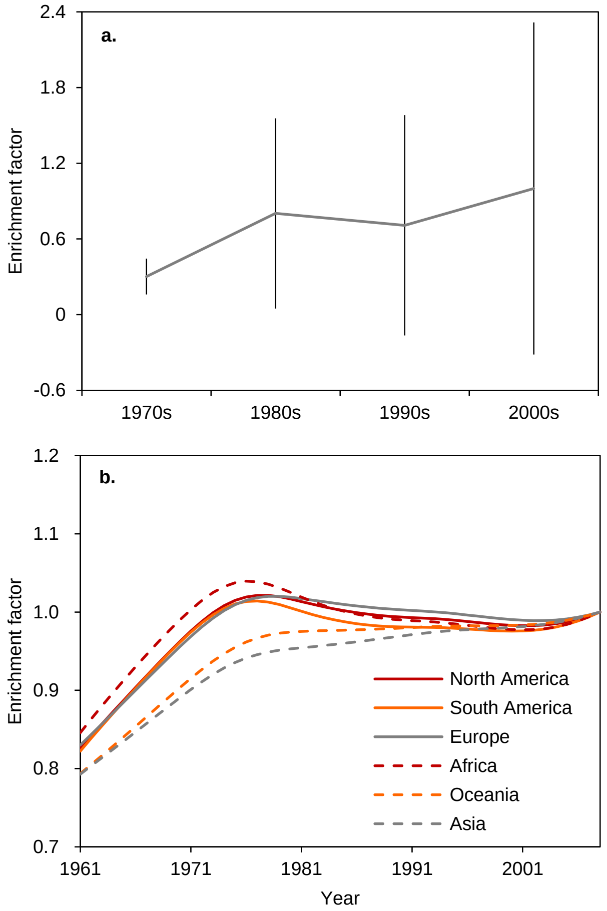

Supplementary Figure 16. Comparison of enrichment factors of THg different periods. Panel **a**: enrichment factors of THg in rice grain relative to 2000s calculated from measurement; and panel **b**: enrichment factors of THg in soil relative to 2008 generated from a global Hg model (see Methods).

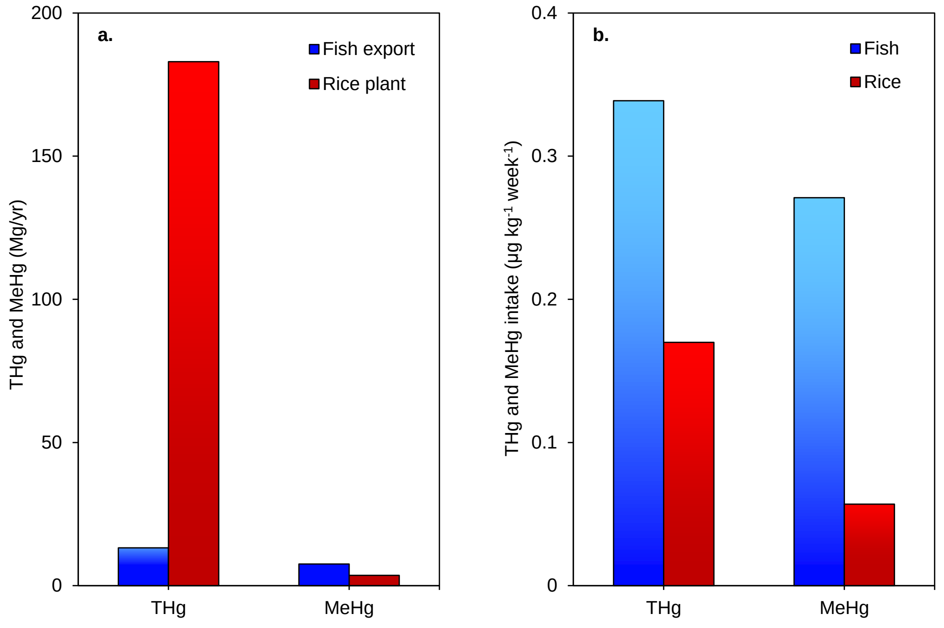

Supplementary Figure 17. Comparison of Hg biotransports between global rice cultivation and fishery (panel a) and inhabitant Hg exposures through consumption of rice and fish (panel b). Data of Hg biotransport through global fishery and inhabitant Hg exposure through fish consumption were referred to published literature[1,2].

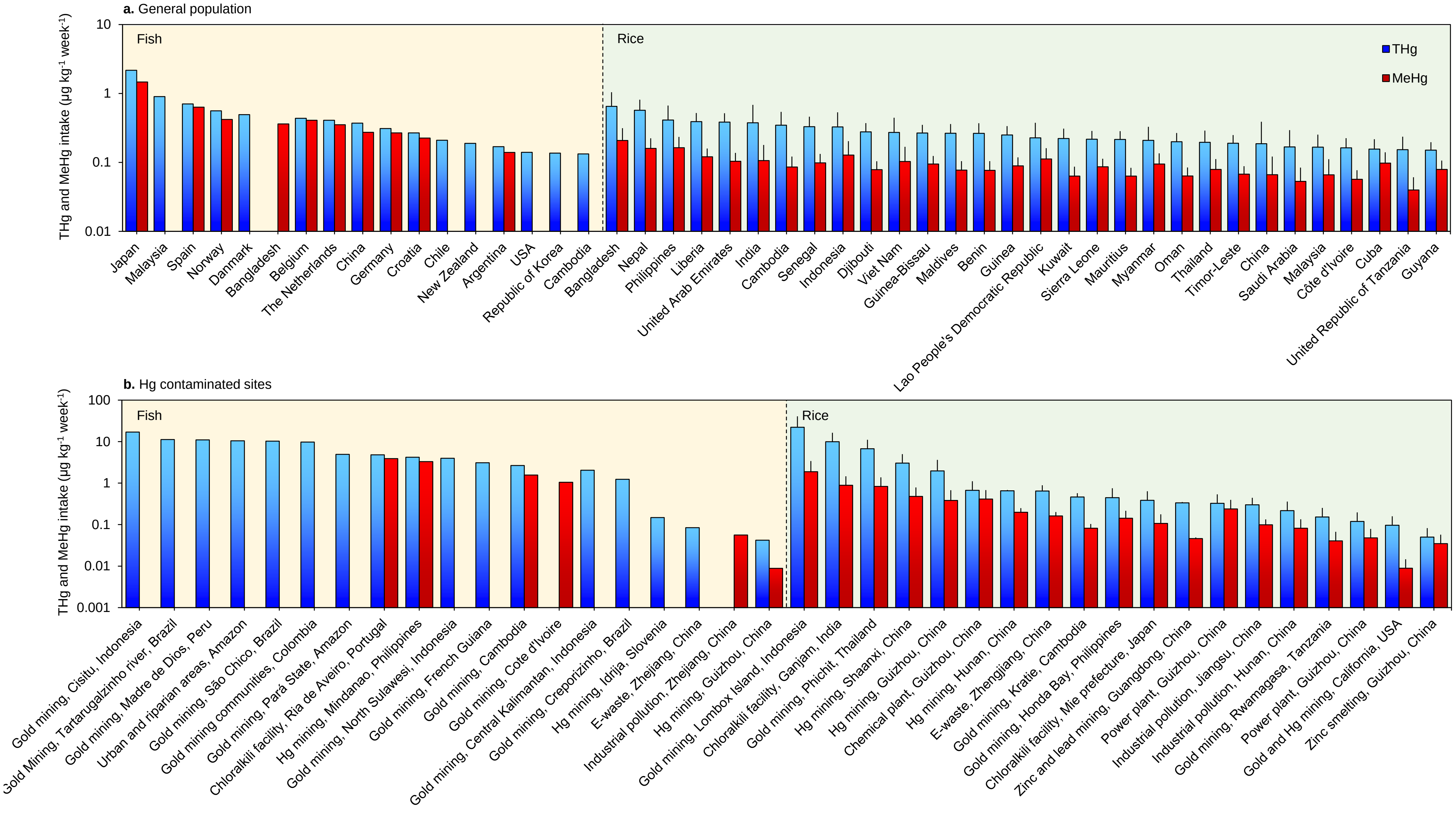

a. General population
Fish
Rice
THg
MeHg
THg and MeHg intake (μg kg-1 week-1)
10
1
0.1
0.01
Japan
Malaysia
Spain
Norway
Danmark
Bangladesh
Belgium
The Netherlands
China
Germany
Croatia
Chile
New Zealand
Argentina
USA
Republic of Korea
Cambodia
Bangladesh
Nepal
Philippines
Liberia
United Arab Emirates
India
Cambodia
Senegal
Indonesia
Djibouti
Viet Nam
Guinea-Bissau
Maldives
Benin
Guinea
Lao People's Democratic Republic
Kuwait
Sierra Leone
Mauritius
Myanmar
Oman
Thailand
Timor-Leste
China
Saudi Arabia
Malaysia
Côte d'Ivoire
Cuba
United Republic of Tanzania
Guyana
b. Hg contaminated sites
Fish
Rice
THg and MeHg intake (μg kg-1 week-1)
100
10
1
0.1
0.01
0.001
Gold mining, Cisitu, Indonesia
Gold Mining, Tartarugalzinho river, Brazil
Gold mining, Madre de Dios, Peru
Urban and riparian areas, Amazon
Gold mining, São Chico, Brazil
Gold mining communities, Colombia
Gold mining, Pará State, Amazon
Chloralkili facility, Ria de Aveiro, Portugal
Hg mining, Mindanao, Philippines
Gold mining, North Sulawesi, Indonesia
Gold mining, French Guiana
Gold mining, Cambodia
Gold mining, Cote d'Ivoire
Gold mining, Central Kalimantan, Indonesia
Gold mining, Creporizinho, Brazil
Hg mining, Idrija, Slovenia
E-waste, Zhejiang, China
Industrial pollution, Zhejiang, China
Hg mining, Guizhou, China
Gold mining, Lombox Island, Indonesia
Chloralkili facility, Ganjam, India
Gold mining, Phichit, Thailand
Hg mining, Shaanxi, China
Hg mining, Guizhou, China
Chemical plant, Guizhou, China
Hg mining, Hunan, China
E-waste, Zhengjiang, China
Gold mining, Kratie, Cambodia
Gold mining, Honda Bay, Philippines
Chloralkili facility, Mie prefecture, Japan
Zinc and lead mining, Guangdong, China
Power plant, Guizhou, China
Industrial pollution, Jiangsu, China
Industrial pollution, Hunan, China
Gold mining, Rwamagasa, Tanzania
Power plant, Guizhou, China
Gold and Hg mining, California, USA
Zinc smelting, Guizhou, China

Supplementary Figure 18. Comparison of inhabitant Hg exposures through consumption of rice and fish in different countries and Hg-contaminated regions. Panel **a**: Hg exposures through consumption of rice and fish in different countries; and panel **b**: Hg exposures through consumption of rice and fish in different Hg-contaminated regions. Data of inhabitant Hg exposure through fish consumption in different countries and Hg-contaminated regions were referred to published literature (see Supplementary Table 9).

Supplementary Note 1

Comparison of mercury exposure via the consumption of rice and fish in different countries and contaminated regions

Globally, 180 (interquartile range of 93 to 410 from the Monte Carlo simulation) Mg of total mercury (THg) and 3.5 (1.8 to 7.0) Mg of methylmercury (MeHg) were generated in rice plants (including rice grains and residues) from terrestrial ecosystems in 2016. In contrast, 13 Mg of THg (including the edible and inedible fractions in seafood) and 7.6 Mg of MeHg were exported from the global ocean via marine fisheries in 2014 (Supplementary Figure 17a)[1]. Moreover, the average human THg and MeHg intake rates contributed by rice consumption were 0.17 (0.11 to 0.28) and 0.057 (0.053 to 0.080) μg kg^{-1} $week^{-1}$ (per capita weekly intake) in 2013, equal to 50% and 21% of that contributed by fish consumption, respectively (Supplementary Figure 17b)[2]. Thus, agricultural cultivation induces a substantial amount of Hg that is generated in rice plants, and rice could be a significant global dietary source of human Hg exposure.

We further compared MeHg exposure between fish and rice in different countries, as well as in different Hg-contaminated and rice-consumed regions, based on published literature (Supplementary Figure 18 and Table 9). According to the existing literature, we found that the inhabitants of Japan potentially faced the highest exposure to Hg via fish consumption in the world (including marine and freshwater fish, 2.1 μg kg^{-1} $week^{-1}$ of THg), followed by the inhabitants of Malaysia and Spain (0.90 and 0.70 μg kg^{-1} $week^{-1}$ of THg, respectively)[3-5]. This situation occurred mainly due to the relatively high fish consumption rates in those countries[6]. Inhabitant Hg exposure via fish consumption was not low in Bangladesh or China, with levels of 0.36 and 0.27 μg MeHg kg^{-1} $week^{-1}$, respectively (Supplementary Figure 18a)[7-9]. Moreover, most of the inhabitants in Bangladesh, China and Malaysia select rice as a staple food. Thus, inhabitant Hg exposure via rice consumption in those three countries was relatively high, with levels of 0.61, 0.19 and 0.17 μg kg^{-1} $week^{-1}$ of THg in 2013, respectively; the values for MeHg were 0.21, 0.066 and 0.066 μg kg^{-1} $week^{-1}$, respectively (Supplementary Figure 18a). This finding means that inhabitant MeHg exposure via the

joint consumption of fish and rice in Malaysia, Bangladesh and China could reach approximately 1.1, 0.57 and 0.34 μg kg^{-1} $week^{-1}$, respectively, 4.1-, 2.1- and 1.3-folds greater than the globe average global value, which only considers the contribution of fish consumption (Supplementary Figure 17b). Previous Hg exposure research has mainly focused on the U.S. and China and countries in Europe and the Amazon region, and fish consumption was considered the single significant dietary source of Hg[5,8,10-14]. Among different regions, Hg exposure via fish consumption is generally high in countries in Europe, America and Asia[4,5,7,9]. In addition to East Asia, exposure via rice consumption is also high in many countries in South and Southeast Asia (Supplementary Figure 18a), and this result has not been found before. Thus, we suggest that future research should consider the joint ingestion of rice and fish in Hg exposure assessments, especially for countries in Southeast and South Asia.

Here, we show that rice consumption could be a substantial dietary Hg exposure source in special areas where rice is a staple food and is cultivated in Hg-contaminated soil. The inhabitants in a gold mining area on Lombox Island (Indonesia) potentially face the highest THg exposure risk via rice consumption, where the THg intake rate via rice consumption could reach 22 μg kg^{-1} $week^{-1}$, followed by a chloralkili facility in Ganjam, India, and a gold mining area in Phichit, Thailand (9.9 and 6.7, respectively, Supplementary Figure 18b). These values are greater than the global general population value by factors of 130, 58 and 40, respectively. As we showed in the main text, MeHg exposure via rice consumption was also high in these Hg-contaminated regions. Additionally, the inhabitant THg exposure via fish consumption was highest in a gold mining area in Cisitu, Indonesia, reaching 17 μg kg^{-1} $week^{-1}$, greater than the global average value by a factor of 50 (Supplementary Figure 18b)[15]. Moreover, high levels of THg exposure via fish consumption were also previously identified in gold mining areas in North Sulawesi and Central Kalimantan, Indonesia, where the intake rates of THg could reach 4.0 and 2.1 μg kg^{-1} $week^{-1}$, higher than the global average value by factors of 12 and 6.2, respectively (Supplementary Figure 18b)[16]. The situations in some Hg-contaminated regions in Cambodia and the Philippines were similar to that in

Indonesia (Supplementary Figure 18b)[17,18], primarily due to the heavy Hg contamination in those regions and the high consumption rates of rice and fish in Southeast Asia[6]. Thus, Hg exposure via the joint consumption of fish and rice is an emerging health issue in Hg-contaminated areas in Southeast Asia.

Overall, inhabitant Hg exposure via fish consumption in Hg-contaminated regions was high in the Amazon region, for instance, in gold mining areas in the Tartarugalzinho River Basin (Brazil) and Madre de Dios (Peru), and in many artisanal gold mining communities in Colombia (Supplementary Figure 18b). The THg intake rates via fish consumption in these contaminated regions were previously found to be 11, 11 and 9.8 $\mu g\ kg^{-1}\ week^{-1}$, respectively[14,19,20], 33, 33 and 28 times greater than the global average value (Supplementary Figure 17b). Many other gold mining areas with high THg intake rates by local inhabitants via fish consumption were also previously identified, such as gold mining areas in São Chico, Pará state, and Creporizinho, Brazil (Supplementary Figure 18b)[21,22]. Although outside the scope of the present study, we suggest that Hg exposure via fish consumption in gold mining areas is a great health challenge in the Amazon. In contrast, Hg exposure via rice consumption in countries in the Amazon region was not high.

Although many Hg-contaminated regions have been identified in China, inhabitant Hg exposure via fish consumption was not high, such as the Hg mining area in Guizhou, China (0.042 and 0.0088 $\mu g\ kg^{-1}\ week^{-1}$ of THg and MeHg, respectively, Supplementary Figure 18b)[23]. This situation probably occurred due to the high fraction of farm-raised fish that are consumed in China, as these fish have a relatively low MeHg concentration[8]. Thus, as suggested by previous studies, rice, but not fish, might be the primary dietary MeHg exposure source in many Hg-contaminated regions in inland China (Supplementary Figure 18b)[23-25].

Supplementary References

1 Lavoie, R. A., Bouffard, A., Maranger, R. & Amyot, M. Mercury transport and human exposure from global marine fisheries. *Sci Rep* **8**, 6705 (2018).

2 Holmes, P., ., James, K. A. F. & Levy, L. S. Is low-level environmental mercury exposure of concern to human health? *Sci. Total Environ.* **408**, 171-182 (2009).

3 Harada, M. Minamata disease: methylmercury poisoning in Japan caused by environmental pollution. *Crit. Rev. Toxicol.* **25**, 1-24 (1995).

4 Hajeb, P. *et al.* Assessment of mercury level in commonly consumed marine fishes in Malaysia. *Food Control* **20**, 79-84 (2009).

5 Bosnir, J., Puntarić, D., Smit, Z. & Capuder, Z. Fish as an indicator of eco-system contamination with mercury. *Croat. Med. J.* **40**, 546-549 (1999).

6 FAO. Food and agriculture data. (Fisheries and Aquaculture Department (FAO), web site: www.fao.org/home/en, 2018).

7 Holsbeek, L., ., Das, H. K. & Joiris, C. R. Mercury in human hair and relation to fish consumption in Bangladesh. *Sci. Total Environ.* **186**, 181-188 (1996).

8 Liu, M. *et al.* Impacts of farmed fish consumption and food trade on methylmercury exposure in China. *Environ. Int.* **120**, 333-344 (2018).

9 Agusa, T. *et al.* Mercury contamination in human hair and fish from Cambodia: levels, specific accumulation and risk assessment. *Environ. Pollut.* **134**, 79-86 (2005).

10 Sunderland, E. M., Li, M. & Bullard, K. Decadal Changes in the Edible Supply of Seafood and Methylmercury Exposure in the United States. *Environ. Health Perspect.* **126**, 017006 (2018).

11 Giang, A. & Selin, N. E. Benefits of mercury controls for the United States. *Proc. Natl. Acad. Sci. U. S. A.* **113**, 286-291 (2015).

12 Li, P. *et al.* Rice consumption contributes to low level methylmercury exposure in southern China. *Environ. Int.* **49**, 18-23 (2012).

13 Johansen, P., Mulvad, G., Pedersen, H. S., Hansen, J. C. & Riget, F. Human accumulation of mercury in Greenland. *Sci. Total Environ.* **377**, 173-178 (2007).

14 Diringer, S. E. *et al.* River transport of mercury from artisanal and small-scale gold mining and risks for dietary mercury exposure in Madre de Dios, Peru. *Environ. Sci. Process Impacts* **17**, 478-487 (2015).

15 Bose-O'Reilly, S. *et al.* A preliminary study on health effects in villagers exposed to mercury in a small-scale artisanal gold mining area in Indonesia. *Environ. Res.* **149**, 274-281 (2016).

16 Castilhos, Z. C. *et al.* Mercury contamination in fish from gold mining areas in Indonesia and human health risk assessment. *Sci. Total Environ.* **368**, 320-325 (2006).

17 Appleton, J., Weeks, J., Calvez, J. & Beinhoff, C. Impacts of mercury contaminated mining waste on soil quality, crops, bivalves, and fish in the Naboc River area, Mindanao, Philippines. *Sci. Total Environ.* **354**, 198-211 (2006).

18 Cheng, Z. *et al.* Dietary exposure and risk assessment of mercury via total diet study in Cambodia. *Chemosphere* **92**, 143-149 (2013).

19 BIDONE *et al.* Fish contamination and human exposure to mercury in Tartarugalzinho river, Amapa State, Northern Amazon, Brazil. A screening approach. *Water Air Soil Pollut.* **97**,

9-15 (1997).
20 Miguel, E. D., Clavijo, D., Ortega, M. F. & Gómez, A. Probabilistic meta-analysis of risk from the exposure to Hg in artisanal gold mining communities in Colombia. *Chemosphere* **108**, 183-189 (2014).
21 Zuleica, C. *et al.* Human exposure and risk assessment associated with mercury contamination in artisanal gold mining areas in the Brazilian Amazon. *Environ. Sci. Pollut. Res.* **22**, 11255-11264 (2015).
22 Castilhos, Z. C., Bidone, E. D. & Lacerda, L. D. Increase of the Background Human Exposure to Mercury Through Fish Consumption due to Gold Mining at the Tapajós River Region, Pará State, Amazon. *Bull. Environ. Contam. Toxicol.* **61**, 202-209 (1998).
23 Zhang, H., Feng, X., Larssen, T., Qiu, G. & Vogt, R. D. In inland China, rice, rather than fish, is the major pathway for methylmercury exposure. *Environ. Health Perspect.* **118**, 1183-1188 (2010).
24 Feng, X. *et al.* Human exposure to methylmercury through rice intake in mercury mining areas, Guizhou Province, China. *Environ. Sci. Technol.* **42**, 326-332 (2007).
25 Li, P., Feng, X., Qiu, G., Shang, L. & Wang, S. Mercury exposure in the population from Wuchuan mercury mining area, Guizhou, China. *Sci. Total Environ.* **395**, 72-79 (2008).
26 Al-Saleh, I. & Abduljabbar, M. Heavy metals (lead, cadmium, methylmercury, arsenic) in commonly imported rice grains (Oryza sativa) sold in Saudi Arabia and their potential health risk. *Int. J. Hyg. Environ. Health.* **220**, 1168-1178 (2017).
27 Al-Saleh, I. & Shinwari, N. Report on the levels of cadmium, lead, and mercury in imported rice grain samples. *Biol. Trace Elem. Res.* **83**, 91-96 (2001).
28 Teherani, D. Trace elements analysis in rice. *J. Radioanal. Nucl. Chem.* **117**, 133-143 (1987).
29 Batista, B. L., Nacano, L. R., de Freitas, R., de Oliveira-Souza, V. C. & Barbosa, F. Determination of essential (Ca, Fe, I, K, Mo) and toxic elements (Hg, Pb) in Brazilian rice grains and estimation of reference daily intake. *Pol. J. food Nutr. Sci.* **3**, 129 (2012).
30 Da Silva, M. J., Paim, A. P. S., Pimentel, M. F., Cervera, M. L. & De la Guardia, M. Determination of mercury in rice by cold vapor atomic fluorescence spectrometry after microwave-assisted digestion. *Anal. Chim. Acta* **667**, 43-48 (2010).
31 Nardi, E. P. *et al.* The use of inductively coupled plasma mass spectrometry (ICP-MS) for the determination of toxic and essential elements in different types of food samples. *Food Chem.* **112**, 727-732 (2009).
32 Silva, L. O. B., da Silva, D. G., Leao, D. J., Matos, G. D. & Ferreira, S. L. C. Slurry sampling for the determination of mercury in rice using cold vapor atomic absorption spectrometry. *Food Anal. Meth.* **5**, 1289-1295 (2012).
33 Cheng, Z. *et al.* Dietary exposure and risk assessment of mercury via total diet study in Cambodia. *Chemosphere* **92**, 143-149 (2013).
34 Brombach, C.-C. *et al.* Methylmercury varies more than one order of magnitude in commercial European rice. *Food Chem.* **214**, 360-365 (2017).
35 Lin, H. *A Study on the Establishment of Heavy Metal Tolerance in Soil through the Heavy M etal concentration of crop. Unpub*, M. Sc. Thesis. Research Institute of Soil Science, National Chung Hsing University, Taichung, Taiwan, (1991).
36 Cheng, J., Zhao, W., Wang, Q., Liu, X. & Wang, W. Accumulation of mercury, selenium

and PCBs in domestic duck brain, liver and egg from a contaminated area with an investigation of their redox responses. *Environ. Toxicol. Pharmacol.* **35**, 388-394 (2013).

37 Zhang, H., Feng, X., Larssen, T., Shang, L. & Li, P. Bioaccumulation of methylmercury versus inorganic mercury in rice (Oryza sativa L.) grain. *Environ. Sci. Technol.* **44**, 4499-4504 (2010).

38 Rothenberg, S. E. *et al.* Characterization of mercury species in brown and white rice (Oryza sativa L.) grown in water-saving paddies. *Environ. Pollut.* **159**, 1283-1289 (2011).

39 Fang, Y. *et al.* Concentrations and health risks of lead, cadmium, arsenic, and mercury in rice and edible mushrooms in China. *Food Chem.* **147**, 147-151 (2014).

40 Qian, Y. *et al.* Concentrations of cadmium, lead, mercury and arsenic in Chinese market milled rice and associated population health risk. *Food Control* **21**, 1757-1763 (2010).

41 Liu, H., Wang, L., Zhang, J., Li, G. & Dai, J. Distribution characteristics, bioaccumulation, and sources of mercury in rice at Nansi Lake area, Shandong Province, China. *J. Anim. Plant Sci.* **25**, 114-121 (2015).

42 Meng, B. *et al.* Distribution patterns of inorganic mercury and methylmercury in tissues of rice (Oryza sativa L.) plants and possible bioaccumulation pathways. *J. Agric. Food Chem.* **58**, 4951-4958 (2010).

43 Rothenberg, S. E. *et al.* Environment and genotype controls on mercury accumulation in rice (Oryza sativa L.) cultivated along a contamination gradient in Guizhou, China. *Sci. Total Environ.* **426**, 272-280 (2012).

44 Jiang, S., Shi, C. & Wu, J. Genotypic differences in arsenic, mercury, lead and cadmium in milled rice (Oryza sativa L.). *Int. J. Food Sci. Nutr.* **63**, 468-475 (2012).

45 Huang, Z., Pan, X.-D., Wu, P.-G., Han, J.-L. & Chen, Q. Health risk assessment of heavy metals in rice to the population in Zhejiang, China. *PloS One* **8**, e75007 (2013).

46 Lin, H.-T., Wong, S.-S. & Li, G.-C. Heavy metal content of rice and shellfish in Taiwan. *J. Food Drug Anal.* **12**, 167-174 (2004).

47 Huamain, C., Chunrong, Z., Cong, T. & Yongguan, Z. Heavy metal pollution in soils in China: status and countermeasures. *Ambio*, 130-134 (1999).

48 Cao, H. *et al.* Heavy metals in rice and garden vegetables and their potential health risks to inhabitants in the vicinity of an industrial zone in Jiangsu, China. *J. Environ. Sci.* **22**, 1792-1799 (2010).

49 Feng, X. *et al.* Human exposure to methylmercury through rice intake in mercury mining areas, Guizhou Province, China. *Environ. Sci. Technol.* **42**, 326-332 (2007).

50 Zhang, H., Feng, X., Larssen, T., Qiu, G. & Vogt, R. D. In inland China, rice, rather than fish, is the major pathway for methylmercury exposure. *Environ. Health Perspect.* **118**, 1183-1188 (2010).

51 Meng, B. *et al.* Localization and speciation of mercury in brown rice with implications for pan-Asian public health. *Environ. Sci. Technol.* **48**, 7974-7981 (2014).

52 Hong, C., Yu, X., Liu, J., Cheng, Y. & Rothenberg, S. E. Low-level methylmercury exposure through rice ingestion in a cohort of pregnant mothers in rural China. *Environ. Res.* **150**, 519-527 (2016).

53 Li, P., Feng, X., Qiu, G., Shang, L. & Wang, S. Mercury exposure in the population from Wuchuan mercury mining area, Guizhou, China. *Sci. Total Environ.* **395**, 72-79 (2008).

54 Cheng, J. *et al.* Mercury levels in fisherman and their household members in Zhoushan,

China: impact of public health. *Sci. Total Environ.* **407**, 2625-2630 (2009).
55 Tang, S., Fan, H., Mao, T. & Huang, Z. Methylmercury accumulation in rice plants (Oryza sativa L.) In low Hg areas and the potential health-Risk exposing in extreme micro-environments.
56 Li, P. *et al.* Rice consumption contributes to low level methylmercury exposure in southern China. *Environ. Int.* **49**, 18-23 (2012).
57 Wu, C. *et al.* Soil mercury speciation and accumulation in rice (Oryza sativa L.) grown in wastewater-irrigated farms. *Appl. Geochem.* **89**, 202-209 (2018).
58 Rothenberg, S. E. *et al.* Stable mercury isotopes in polished rice (Oryza Sativa L.) and hair from rice consumers. *Environ. Sci. Technol.* **51**, 6480-6488 (2017).
59 Lin, Y. *et al.* Ultrasensitive speciation analysis of mercury in rice by headspace solid phase microextraction using porous carbons and gas chromatography-dielectric barrier discharge optical emission spectrometry. *Environ. Sci. Technol.* **50**, 2468-2476 (2016).
60 Huang, L., Li, B., Tam, N. F.-Y., Wang, X. & Ye, Z. Effects of environment and genotype on mercury and methylmercury accumulation in rice (Oryza sativa L.). *Plant Soil* **427**, 269-280 (2018).
61 Zhu, C., Shen, G., Yan, Y. & He, J. Genotypic variation in grain mercury accumulation of lowland rice. *J. Plant Nutr. Soil Sci.* **171**, 281-285 (2008).
62 Hang, X. *et al.* Evaluation of Mercury Uptake and Distribution in Rice (Oryza sativa L.). *Bull. Environ. Contam. Toxicol.* **100**, 451-456 (2018).
63 Zhou, J. *et al.* Influence of soil mercury concentration and fraction on bioaccumulation process of inorganic mercury and methylmercury in rice (Oryza sativa L.). *Environ. Sci. Pollut. Res.* **22**, 6144-6154 (2015).
64 Li, Y. Environmental contamination and risk assessment of mercury from a historic mercury mine located in southwestern China. *Environ. Geochem. Health* **35**, 27-36 (2013).
65 Shi, J.-B., Liang, L.-N. & Jiang, G.-B. Simultaneous determination of methylmercury and ethylmercury in rice by capillary gas chromatography coupled on-line with atomic fluorescence spectrometry. *J. AOAC Int.* **88**, 665-669 (2005).
66 Lee, D., Thomas, B., Roughan, J. & Watters, E. Mercury content of some foodstuffs of vegetable origin. *Pestic. Sci.* **3**, 13-17 (1972).
67 Leblanc, J.-C. *et al.* Dietary exposure estimates of 18 elements from the 1st French Total Diet Study. *Food Addit. Contam.* **22**, 624-641 (2005).
68 Sarkar, A., Aronson, K. J., Patil, S. & Hugar, L. B. Emerging health risks associated with modern agriculture practices: A comprehensive study in India. *Environ. Res.* **115**, 37-50 (2012).
69 Srikumar, T. The mineral and trace element composition of vegetables, pulses and cereals of southern India. *Food Chem.* **46**, 163-167 (1993).
70 Singh, J., Upadhyay, S. K., Pathak, R. K. & Gupta, V. Accumulation of heavy metals in soil and paddy crop (Oryza sativa), irrigated with water of Ramgarh Lake, Gorakhpur, UP, India. *Toxicol Environ. Chem.* **93**, 462-473 (2011).
71 Shishido, S. & SUZUKI, T. Estimation of daily intake of inorganic or organic mercury via diet. *Tohoku J. Exp. Med.* **114**, 369-377 (1974).
72 Suzuki, T., Satoh, H., Yamamoto, R. & Kashiwazaki, H. Selenium and mercury in foodstuff from a locality with elevated intake of methylmercury. *Bull. Environ. Contam. Toxicol.* **24**,

805-812 (1980).
73 Lee, H.-S. *et al.* Dietary exposure of the Korean population to arsenic, cadmium, lead and mercury. *J. Food Compos. Anal.* **19**, S31-S37 (2006).
74 Rothenberg, S. E., Mgutshini, N. L., Bizimis, M., Johnson-Beebout, S. E. & Ramanantsoanirina, A. Retrospective study of methylmercury and other metal (loid) s in Madagascar unpolished rice (Oryza sativa L.). *Environ. Pollut.* **196**, 125-133 (2015).
75 Zarcinas, B. A., Ishak, C. F., McLaughlin, M. J. & Cozens, G. Heavy metals in soils and crops in Southeast Asia. *Environ. Geochem. Health* **26**, 343-357 (2004).
76 Appleton, J., Weeks, J., Calvez, J. & Beinhoff, C. Impacts of mercury contaminated mining waste on soil quality, crops, bivalves, and fish in the Naboc River area, Mindanao, Philippines. *Sci. Total Environ.* **354**, 198-211 (2006).
77 Abrahamsson, E. & Ekelund, M. (DiVA portal, 2015).
78 da Silva, D. G., Portugal, L. A., Serra, A. M., Ferreira, S. L. & Cerdà, V. Determination of mercury in rice by MSFIA and cold vapour atomic fluorescence spectrometry. *Food Chem.* **137**, 159-163 (2013).
79 Taylor, H. *et al.* Environmental assessment of mercury contamination from the Rwamagasa artisanal gold mining centre, Geita District, Tanzania. *Sci. Total Environ.* **343**, 111-133 (2005).
80 Zarcinas, B. A., Pongsakul, P., McLaughlin, M. J. & Cozens, G. Heavy metals in soils and crops in Southeast Asia 2. Thailand. *Environ. Geochem. Health* **26**, 359-371 (2004).
81 Bennett, J. P., Chiriboga, E., Coleman, J. & Waller, D. M. Heavy metals in wild rice from northern Wisconsin. *Sci. Total Environ.* **246**, 261-269 (2000).
82 Fu, J. *et al.* High levels of heavy metals in rice (Oryzasativa L.) from a typical E-waste recycling area in southeast China and its potential risk to human health. *Chemosphere* **71**, 1269-1275 (2008).
83 Zhao, G., Zhou, H. & Wang, Z. Concentrations of selected heavy metals in food from four e-waste disassembly localities and daily intake by local residents. *J. Environ. Sci. Health Part A-Toxic/Hazard. Subst. Environ. Eng.* **45**, 824-835 (2010).
84 Feng, X. & Qiu, G. Mercury pollution in Guizhou, Southwestern China—an overview. *Sci. Total Environ.* **400**, 227-237 (2008).
85 Horvat, M. *et al.* Total mercury, methylmercury and selenium in mercury polluted areas in the province Guizhou, China. *Sci. Total Environ.* **304**, 231-256 (2003).
86 Li, B. *et al.* Variations and constancy of mercury and methylmercury accumulation in rice grown at contaminated paddy field sites in three Provinces of China. *Environ. Pollut.* **181**, 91-97 (2013).
87 Meng, M. *et al.* Accumulation of total mercury and methylmercury in rice plants collected from different mining areas in China. *Environ. Pollut.* **184**, 179-186 (2014).
88 Qiu, G. *et al.* Methylmercury accumulation in rice (Oryza sativa L.) grown at abandoned mercury mines in Guizhou, China. *J. Agric. Food Chem.* **56**, 2465-2468 (2008).
89 Qiu, G. *et al.* Environmental geochemistry of an abandoned mercury mine in Yanwuping, Guizhou Province, China. *Environ. Res.* **125**, 124-130 (2013).
90 Rothenberg, S. E., Yu, X. & Zhang, Y. Prenatal methylmercury exposure through maternal rice ingestion: Insights from a feasibility pilot in Guizhou Province, China. *Environ. Pollut.* **180**, 291-298 (2013).

91 Wang, X. *et al.* Multielemental contents of foodstuffs from the Wanshan (China) mercury mining area and the potential health risks. *Appl. Geochem.* **26**, 182-187 (2011).

92 Li, W. C., Ouyang, Y. & Ye, Z. H. Accumulation of mercury and cadmium in rice from paddy soil near a mercury mine. *Environ. Toxicol. Chem.* **33**, 2438-2447 (2014).

93 Cheng, J. *et al.* Mercury pollution in two typical areas in Guizhou province, China and its neurotoxic effects in the brains of rats fed with local polluted rice. *Environ. Geochem. Health* **28**, 499-507 (2006).

94 Qiu, G., Feng, X., Wang, S. & Shang, L. Environmental contamination of mercury from Hg-mining areas in Wuchuan, northeastern Guizhou, China. *Environ. Pollut.* **142**, 549-558 (2006).

95 Qiu, G., Feng, X., Meng, B., Sommar, J. & Gu, C. Environmental geochemistry of an active Hg mine in Xunyang, Shaanxi Province, China. *Appl. Geochem.* **27**, 2280-2288 (2012).

96 Qiu, G., Feng, X., Meng, B. & Wang, X. Methylmercury in rice (Oryza sativa L.) grown from the Xunyang Hg mining area, Shaanxi province, northwestern China. *Pure Appl. Chem.* **84**, 281-289 (2011).

97 Haiyan, W. & Stuanes, A. O. Heavy metal pollution in air-water-soil-plant system of Zhuzhou City, Hunan Province, China. *Water Air Soil Pollut.* **147**, 79-107 (2003).

98 Hang, X. *et al.* Risk assessment of potentially toxic element pollution in soils and rice (Oryza sativa) in a typical area of the Yangtze River Delta. *Environ. Pollut.* **157**, 2542-2549 (2009).

99 Lenka, M., Panda, K. K. & Panda, B. B. Monitoring and assessment of mercury pollution in the vicinity of a chloralkali plant. IV. Bioconcentration of mercury in in situ aquatic and terrestrial plants at Ganjam, India. *Arch. Environ. Contam. Toxicol.* **22**, 195-202 (1992).

100 Krisnayanti, B. D. *et al.* Assessment of environmental mercury discharge at a four-year-old artisanal gold mining area on Lombok Island, Indonesia. *J. Environ. Monit.* **14**, 2598-2607 (2012).

101 Morishita, T., Kishino, K. & Idaka, S. Mercury contamination of solls, rice plants, and human hair in the vicinity of a mercury mine in mie prefecture, Japan. *Soil Sci. Plant Nutr.* **28**, 523-534 (1982).

102 Maramba, N. P. *et al.* Environmental and human exposure assessment monitoring of communities near an abandoned mercury mine in the Philippines: A toxic legacy. *J. Environ. Manage.* **81**, 135-145 (2006).

103 Pataranawat, P., Parkpian, P., Polprasert, C., Delaune, R. & Jugsujinda, A. Mercury emission and distribution: Potential environmental risks at a small-scale gold mining operation, Phichit Province, Thailand. *J. Environ. Sci. Health Part A-Toxic/Hazard. Subst. Environ. Eng.* **42**, 1081-1093 (2007).

104 Windham-Myers, L. *et al.* Mercury cycling in agricultural and managed wetlands: A synthesis of methylmercury production, hydrologic export, and bioaccumulation from an integrated field study. *Sci. Total Environ.* **484**, 221-231 (2014).

105 Abdelhamid, M. T., Horiuchi, T. & Oba, S. Composting of rice straw with oilseed rape cake and poultry manure and its effects on faba bean (Vicia faba L.) growth and soil properties. *Bioresour. Technol.* **93**, 183-189 (2004).

106 Bhattacharyya, P. *et al.* Effects of rice straw and nitrogen fertilization on greenhouse gas emissions and carbon storage in tropical flooded soil planted with rice. *Soil Tillage Res.*

124, 119-130 (2012).
107 Koopmans, A. & Koppejan, J. Agricultural and forest residues-generation, utilization and availability. *Paper presented at the regional consultation on modern applications of biomass energy* **6**, 10 (1997).
108 Wang, X., Lu, X., Li, F. & Yang, G. Effects of temperature and carbon-nitrogen (C/N) ratio on the performance of anaerobic co-digestion of dairy manure, chicken manure and rice straw: focusing on ammonia inhibition. *PloS One* **9**, e97265 (2014).
109 Zhu, N. Effect of low initial C/N ratio on aerobic composting of swine manure with rice straw. *Bioresour. Technol.* **98**, 9-13 (2007).
110 Devêvre, O. C. & Horwáth, W. R. Decomposition of rice straw and microbial carbon use efficiency under different soil temperatures and moistures. *Soil Biol. Biochem.* **32**, 1773-1785 (2000).
111 Verma, T. & Bhagat, R. Impact of rice straw management practices on yield, nitrogen uptake and soil properties in a wheat-rice rotation in northern India. *Fertil. Res.* **33**, 97-106 (1992).
112 Zhang, R. & Zhang, Z. Biogasification of rice straw with an anaerobic-phased solids digester system. *Bioresour. Technol.* **68**, 235-245 (1999).
113 Dobermann, A. & Fairhurst, T. Rice straw management. *Better Crop Int.* **16**, 7-11 (2002).
114 Tsai, W., Lee, M. & Chang, Y. Fast pyrolysis of rice straw, sugarcane bagasse and coconut shell in an induction-heating reactor. *J. Anal. Appl. Pyrolysis* **76**, 230-237 (2006).
115 He, Y., Pang, Y., Liu, Y., Li, X. & Wang, K. Physicochemical characterization of rice straw pretreated with sodium hydroxide in the solid state for enhancing biogas production. *Energy Fuels* **22**, 2775-2781 (2008).
116 Miura, Y. & Kanno, T. Emissions of trace gases (CO2, CO, CH4, and N2O) resulting from rice straw burning. *Soil Sci. Plant Nutr.* **43**, 849-854 (1997).
117 Pütün, A. E., Apaydın, E. & Pütün, E. Rice straw as a bio-oil source via pyrolysis and steam pyrolysis. *Energy* **29**, 2171-2180 (2004).
118 Bird, J., Pettygrove, G. & Eadie, J. The impact of waterfowl foraging on the decomposition of rice straw: mutual benefits for rice growers and waterfowl. *J. Appl. Ecol.* **37**, 728-741 (2000).
119 Gu, Y., Chen, X., Liu, Z., Zhou, X. & Zhang, Y. Effect of inoculum sources on the anaerobic digestion of rice straw. *Bioresour. Technol.* **158**, 149-155 (2014).
120 Chandra, R., Takeuchi, H. & Hasegawa, T. Hydrothermal pretreatment of rice straw biomass: a potential and promising method for enhanced methane production. *Appl. Energy* **94**, 129-140 (2012).
121 Rashad, F. M., Saleh, W. D. & Moselhy, M. A. Bioconversion of rice straw and certain agro-industrial wastes to amendments for organic farming systems: 1. Composting, quality, stability and maturity indices. *Bioresour. Technol.* **101**, 5952-5960 (2010).
122 Jhorar, B., Phogat, V. & Malik, R. Kinetics of composting rice straw with glue waste at different carbon: nitrogen ratios in a semiarid environment. *Arid Land Res. Manag.* **5**, 297-306 (1991).
123 Tang, S., Fan, H., Mao, T. & Huang, Z. Methylmercury accumulation in rice plants (Oryza sativa L.) In low Hg areas and the potential health-Risk exposing in extreme micro-environments. *Rev. Adv. Mater. Sci.* **2**, 1-6 (2017).

124 Feng, X., Li, G. & Qiu, G. A preliminary study on mercury contamination to the environment from artisanal zinc smelting using indigenous methods in Hezhang county, Guizhou, China—Part 1: mercury emission from zinc smelting and its influences on the surface waters. *Atmos. Environ.* **38**, 6223-6230 (2004).

125 Liu, W.-X., Shen, L.-F., Liu, J.-W., Wang, Y.-W. & Li, S.-R. Uptake of toxic heavy metals by rice (Oryza sativa L.) cultivated in the agricultural soil near Zhengzhou City, People's Republic of China. *Bull. Environ. Contam. Toxicol.* **79**, 209-213 (2007).

126 Zhang, H. *et al.* Selenium in soil inhibits mercury uptake and translocation in rice (Oryza sativa L.). *Environ. Sci. Technol.* **46**, 10040-10046 (2012).

127 Zhao, J. *et al.* Selenium modulates mercury uptake and distribution in rice (Oryza sativa L.), in correlation with mercury species and exposure level. *Metallomics* **6**, 1951-1957 (2014).

128 Meng, B. *et al.* Inorganic mercury accumulation in rice (Oryza sativa L.). *Environ. Toxicol. Chem.* **31**, 2093-2098 (2012).

129 Li, Y. *et al.* Thiosulfate amendment reduces mercury accumulation in rice (Oryza sativa L.). *Plant Soil* **430**, 413-422 (2018).

130 Yin, R., Feng, X. & Meng, B. Stable mercury isotope variation in rice plants (Oryza sativa L.) from the Wanshan mercury mining district, SW China. *Environ. Sci. Technol.* **47**, 2238-2245 (2013).

131 Zhao, H. *The preliminary analysis of the distribution of total mercury in rice (Oryza Sativa L.) from the main rice producing areas of China* Master's thesis thesis, Guizhou Normal University, (2017).

132 Zheng, S., Tang, J., Zheng, H., Xue, Y. & Zheng, X. Pollution characteristics and risk assessments of mercury in wastewater-irrigated paddy fields. *Chin. Environ. Sci.* **35**, 2729-2736 (2015).

133 Jiang, X. & Zhu, Y. Mercury migration in soil - rice system. *Chongqing Environ. Sci.* **17**, 54-56 (1995).

134 BA, S. *Handbook of energy for world agric. New York.* 504 (Elsevier Science, 1990).

135 Lal, R. World crop residues production and implications of its use as a biofuel. *Environ. Int.* **31**, 575-584 (2005).

136 Turn, S. *et al.* Elemental characterization of particulate matter emitted from biomass burning: Wind tunnel derived source profiles for herbaceous and wood fuels. *J. Geophys. Res.-Atmos.* **102**, 3683-3699 (1997).

137 Obrist, D., Moosmüller, H., Schürmann, R., Chen, L.-W. A. & Kreidenweis, S. M. Particulate-phase and gaseous elemental mercury emissions during biomass combustion: controlling factors and correlation with particulate matter emissions. *Environ. Sci. Technol.* **42**, 721-727 (2007).

138 Yuan, L. *et al.* Responses of rice production, milled rice quality and soil properties to various nitrogen inputs and rice straw incorporation under continuous plastic film mulching cultivation. *Field Crop. Res.* **155**, 164-171 (2014).

139 Gadde, B., Bonnet, S., Menke, C. & Garivait, S. Air pollutant emissions from rice straw open field burning in India, Thailand and the Philippines. *Environ. Pollut.* **157**, 1554-1558 (2009).

140 Holmes, P., ., James, K. A. F. & Levy, L. S. Is low-level environmental mercury exposure

of concern to human health? *Sci. Total Environ.* **408**, 171-182 (2009).
141 Harada, M. Minamata disease: methylmercury poisoning in Japan caused by environmental pollution. *Crit. Rev. Toxicol.* **25**, 1-24 (1995).
142 Hajeb, P. *et al.* Assessment of mercury level in commonly consumed marine fishes in Malaysia. *Food Control* **20**, 79-84 (2009).
143 Bosnir, J., Puntarić, D., Smit, Z. & Capuder, Z. Fish as an indicator of eco-system contamination with mercury. *Croat. Med. J.* **40**, 546-549 (1999).
144 Mangerud, G. Dietary Mercury Exposure in Selected Norwegian Municipalities: The Norwegian Fish and Game Study, Part C. Available: http://www.nhv.se/upload/dokument/forskning/Publikationer/MPH/MPH%202005-202002%202020GMangerud.pdf (2005).
145 Johansen, P., Mulvad, G., Pedersen, H. S., Hansen, J. C. & Riget, F. Human accumulation of mercury in Greenland. *Sci. Total Environ.* **377**, 173-178 (2007).
146 Holsbeek, L., ., Das, H. K. & Joiris, C. R. Mercury in human hair and relation to fish consumption in Bangladesh. *Sci. Total Environ.* **186**, 181-188 (1996).
147 Liu, M. *et al.* Impacts of farmed fish consumption and food trade on methylmercury exposure in China. *Environ. Int.* **120**, 333-344 (2018).
148 Sandra, C. & Antonia, F. Mercury content in Chilean fish and estimated intake levels. *Food Addit. Contam.* **24**, 955-959 (2007).
149 Vannoort, R. & Cressey, P. 1997/98 New Zealand Total Diet Survey: Part 2-Elements. (New Zealand Ministry of Health, 2000).
150 Sunderland, E. M. Mercury exposure from domestic and imported estuarine and marine fish in the U.S. seafood market. *Environ. Health Perspect.* **115**, 235-242 (2007).
151 Agusa, T. *et al.* Mercury contamination in human hair and fish from Cambodia: levels, specific accumulation and risk assessment. *Environ. Pollut.* **134**, 79-86 (2005).
152 Bose-O'Reilly, S. *et al.* A preliminary study on health effects in villagers exposed to mercury in a small-scale artisanal gold mining area in Indonesia. *Environ. Res.* **149**, 274-281 (2016).
153 BIDONE *et al.* Fish contamination and human exposure to mercury in Tartarugalzinho river, Amapa State, Northern Amazon, Brazil. A screening approach. *Water Air Soil Pollut.* **97**, 9-15 (1997).
154 Diringer, S. E. *et al.* River transport of mercury from artisanal and small-scale gold mining and risks for dietary mercury exposure in Madre de Dios, Peru. *Environ. Sci. Process Impacts* **17**, 478-487 (2015).
155 Passos, C. J. & Mergler, D. Human mercury exposure and adverse health effects in the Amazon: a review. *Cadernos De Saúde Pública* **24**, s503 (2008).
156 Zuleica, C. *et al.* Human exposure and risk assessment associated with mercury contamination in artisanal gold mining areas in the Brazilian Amazon. *Environ. Sci. Pollut. Res.* **22**, 11255-11264 (2015).
157 Miguel, E. D., Clavijo, D., Ortega, M. F. & Gómez, A. Probabilistic meta-analysis of risk from the exposure to Hg in artisanal gold mining communities in Colombia. *Chemosphere* **108**, 183-189 (2014).
158 Castilhos, Z. C., Bidone, E. D. & Lacerda, L. D. Increase of the Background Human Exposure to Mercury Through Fish Consumption due to Gold Mining at the Tapajós River

Region, Pará State, Amazon. *Bull. Environ. Contam. Toxicol.* **61**, 202-209 (1998).

159 Cláudia Leopoldina, M., Mário, P., Maria Eduarda, P. & Armando Costa, D. Mercury distribution in key tissues of fish (Liza aurata) inhabiting a contaminated estuary-implications for human and ecosystem health risk assessment. *J. Environ. Monit.* **11**, 1004-1012 (2009).

160 Castilhos, Z. C. *et al.* Mercury contamination in fish from gold mining areas in Indonesia and human health risk assessment. *Sci. Total Environ.* **368**, 320-325 (2006).

161 Fréry, N., . *et al.* Gold-mining activities and mercury contamination of native amerindian communities in French Guiana: key role of fish in dietary uptake. *Environ. Health Perspect.* **109**, 449-456 (2001).

162 Mason, R. P. *et al.* An assessment of the impact of artisanal and commercial gold mining on mercury and methylmercury levels in the environment and fish in Cote d'Ivoire. *Sci. Total Environ.* **665**, 1158-1167.

163 Kobal, A. B. *et al.* Exposure to mercury in susceptible population groups living in the former mercury mining town of Idrija, Slovenia. *Environ. Res.* **152**, 434-445 (2017).

164 Liang, P. *et al.* Human exposure to mercury in a compact fluorescent lamp manufacturing area: By food (rice and fish) consumption and occupational exposure. *Environ. Pollut.* **198**, 126-132 (2015).
