# Supplementary material for: Rice life cycle-based global mercury biotransport and human methylmercury exposure
Source: Nat Commun. 2019 Nov 14;10:5164. doi: 10.1038/s41467-019-13221-2 (PMC6856186; doi:10.1038/s41467-019-13221-2)
Supplement: Supplementary file 2 — Description of Additional Supplementary Files [file 41467_2019_13221_MOESM2_ESM.pdf]

Description of Additional Supplementary Files

File Name: Supplementary Data 1
Description: Material flows of total mercury (THg) in rice grains and residues in different regions across the world

File Name: Supplementary Data 2
Description: Material flows of methylmercury (MeHg) in rice grains and residues in different regions across the world

File Name: Supplementary Data 3
Description: Material flows of MeHg in rice grains and residues, and inhabitants THg and MeHg exposure through rice consumption in different countries and regions across the world

File Name: Supplementary Data 4
Description: Concentrations of THg and MeHg in rice grains in different regions Supplementary Data 5. Total nitrogen contained in rice residues

File Name: Supplementary Data 6
Description: Concentrations of THg in different parts of rcie plants

File Name: Supplementary Data 7
Description: Dry weight ratio of straw to grain for different crops

File Name: Supplementary Data 8
Description: Moisture content in rice residues

File Name: Supplementary Data 9
Description: Inhabitant THg and MeHg exposure through fish consumption in different countries and contaminated regions in the world

File Name: Supplementary Data 10
Description: Amounts of THg embodied in international trades of rice based on reports of export countries and regions

File Name: Supplementary Data 11
Description: Amounts of THg embodied in international trades of rice based on reports of import countries and regions

File Name: Supplementary Data 12
Description: Amounts of MeHg embodied in international trades of rice based on reports of export countries and regions

File Name: Supplementary Data 13
Description: Amounts of MeHg embodied in international trades of rice based on reports of import countries and regions

File Name: Supplementary Data 14
Description: Amounts of THg and MeHg embodied in international trades of rice between different regions across the world

File Name: Supplementary Data 15
Description: Potential THg and MeHg exposure through consumption of rice from Hg-contaminated sites and per-foetus intelligence quotient decrements

File Name: Supplementary Data 16
Description: Global THg generated in crop residues in 2016

File Name: Supplementary Data 17
Description: Uncertainties of biotransports of THg and MeHg from production to consumption of rice grain, and generated in rice residues

File Name: Supplementary Data 18
Description: Uncertainties of THg and MeHg generated in rice grain and residues and health impacts in different countries and regions

File Name: Supplementary Data 19
Description: Enrichment factors of THg in soil relative to the year 2008
